# Whole-Exome Sequencing of Congenital Glaucoma Patients Reveals Hypermorphic Variants in *GPATCH3*, a New Gene Involved in Ocular and Craniofacial Development

**DOI:** 10.1038/srep46175

**Published:** 2017-04-11

**Authors:** Jesús-José Ferre-Fernández, José-Daniel Aroca-Aguilar, Cristina Medina-Trillo, Juan-Manuel Bonet-Fernández, Carmen-Dora Méndez-Hernández, Laura Morales-Fernández, Marta Corton, María-José Cabañero-Valera, Marta Gut, Raul Tonda, Carmen Ayuso, Miguel Coca-Prados, Julián García-Feijoo, Julio Escribano

**Affiliations:** 1 Área de Genética, Facultad de Medicina/Instituto de Investigación en Discapacidades Neurológicas (IDINE), Universidad de Castilla-La Mancha, Albacete, Spain; 2 Cooperative Research Network on Age-Related Ocular Pathology, Visual and Life Quality, Instituto de Salud Carlos III, Madrid, Spain; 3 Servicio de Oftalmología, Hospital San Carlos, Madrid, Spain/Instituto de Investigación Sanitaria del Hospital Clínico San Carlos, Madrid, Spain; 4 Área de Genética & Genómica, Instituto de Investigación Sanitaria-Hospital Universitario Fundación Jiménez Díaz-Universidad Autónoma de Madrid (IIS-FJD, UAM)/Centro de Investigación Biomédica en Red de Enfermedades Raras (CIBERER), Madrid, Spain; 5 CNAG-CRG, Centre for Genomic Regulation (CRG), Institute of Science and Technology (BIST), Centre for Genomic Analysis (CNAG), Barcelona, Spain; 6 Universitat Pompeu Fabra (UPF), Barcelona, Spain; 7 Fundación de Investigación Oftalmológica, Instituto Oftalmológico Fernandez-Vega, Oviedo, Spain; 8 Department of Ophthalmology and Visual Science, Yale University School of Medicine, New Haven, CT, USA

## Abstract

Congenital glaucoma (CG) is a heterogeneous, inherited and severe optical neuropathy that originates from maldevelopment of the anterior segment of the eye. To identify new disease genes, we performed whole-exome sequencing of 26 unrelated CG patients. In one patient we identified two rare, recessive and hypermorphic coding variants in *GPATCH3*, a gene of unidentified function, and 5% of a second group of 170 unrelated CG patients carried rare variants in this gene. The recombinant GPATCH3 protein activated *in vitro* the proximal promoter of *CXCR4,* a gene involved in embryo neural crest cell migration. The GPATCH3 protein was detected in human tissues relevant to glaucoma (e.g., ciliary body). This gene was expressed in the dermis, skeletal muscles, periocular mesenchymal-like cells and corneal endothelium of early zebrafish embryos. Morpholino-mediated knockdown and transient overexpression of *gpatch3* led to varying degrees of goniodysgenesis and ocular and craniofacial abnormalities, recapitulating some of the features of zebrafish embryos deficient in the glaucoma-related genes *pitx2 and foxc1*. In conclusion, our data suggest the existence of high genetic heterogeneity in CG and provide evidence for the role of *GPATCH3* in this disease. We also show that *GPATCH3* is a new gene involved in ocular and craniofacial development.

Identification of the molecular genetic basis of primary congenital glaucoma (PCG) may improve clinicians’ ability to diagnose, counsel, treat, and manage their patients. PCG is the most common non-syndromic glaucoma in infancy. It represents an important cause of visual loss in children and has been reported to occur in 1/5000 to 1/10000 births in Western countries^1^. This condition usually manifests within the first year of life with the classic symptoms of tearing, photophobia and clouding of the cornea^2^. The pathogenesis of PCG remains uncertain but it is thought to originate from the isolated maldevelopment of structures in the anterior segment of the eye due to arrested maturation of tissues derived from cranial neural crest cells^3^,^4^. This anomaly leads to decreased aqueous humor outflow. Although PCG can be transmitted according to different modes of inheritance, autosomal recessive transmission of *CYTOCHROME P450, SUBFAMILY I, POLYPEPTIDE 1* [*CYP1B1*, Mendelian Inheritance in Man (MIM): 601771] mutations has been well documented in a large proportion of cases^5^,^6^,^7^,^8^,^9^. *MYOCILIN (MYOC*, MIM: 601652)^10^,^11^ and *FORKHEAD BOX C1*^10^,^12^,^13^ (*FOXC1,* MIM: 601090) alterations have also been found in small numbers of PCG patients. Genetic and phenotypic variability is a hallmark of this disease. Variable iridocorneal angle anomalies ranging from severe (characterized by agenesis of Schlemm’s canal) to mild gonyodysgenesis have been found even in *CYP1B1* associated PCG^14^, which suggests the role of modifier factors in the phenotypic outcome. Our group has reported that approximately 30% of Spanish PCG patients carry loss-of-function *CYP1B1* variants, with most of these variants resulting in null genotypes^6^. Even among the cases with null enzymatic activity, remarkable phenotypic variation is present^6^,^7^,^15^. These facts, along with the existence of incomplete penetrance and the discovery of a significant proportion of patients who carry non-dominant heterozygous *CYP1B1* mutations^6^, suggest that more than one gene is involved in PCG. The genetic complexity of this developmental disease has hampered the identification of new glaucoma genes by conventional linkage analysis. In this study, we carried out whole-exome sequencing (WES) and comprehensive functional evaluations to identify new candidate genes. Our results suggest the existence of remarkable genetic heterogeneity in congenital glaucoma and provide evidence for the role of *GPATCH3* in this disease. We also show that *GPATCH3* is a new gene involved in ocular and craniofacial development.

## Results

### Identification of rare *GPATCH3* variants by WES

**Figure 1:**
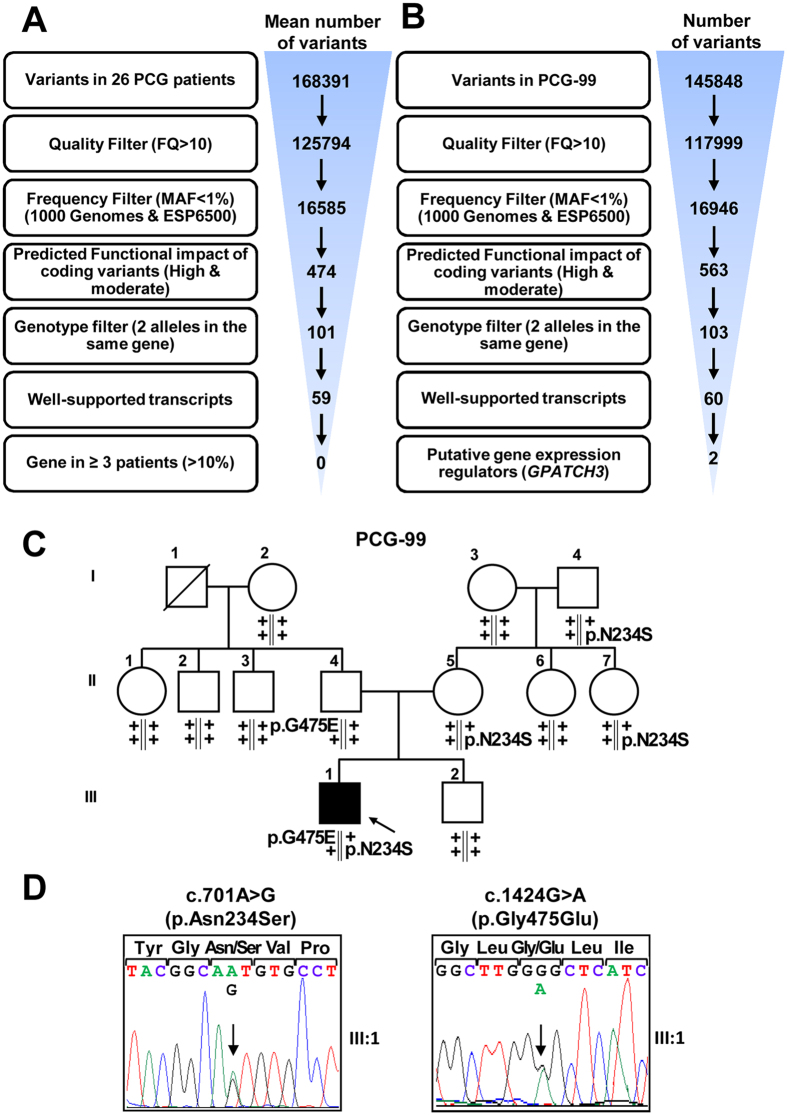
Variant filtering schemes used for variant prioritization of exome data and pedigree analysis of *GPATCH3* variants. (**A**) The exome was interrogated for variants present in genes shared by at least three patients (approximately 10% of patients) under an autosomal recessive model in the discovery group. Well-supported transcripts: transcripts with curated RefSeq records (identified with the prefix “NM_” in GeneBank) and present in the Consensus Coding Sequence database. (**B**) Filtering steps followed to identify candidate variants in genes involved in gene expression regulation and present in only one patient (PCG-99). (**C**) Candidate variant segregation in family PCG99. The oblique lines represent dead subjects. The black symbol indicates primary congenital glaucoma. The arrow in the pedigree shows the index case. +: wild-type allele. The vertical lines below the symbols represent chromosomes. (**D**) Electropherograms of the *GPATCH3* variants identified in family PCG-99. Both mutations were detected in the heterozygous state. Arrows indicate the location of mutations.

The sample selected for WES in this study included a subset of 26 severe PCG cases from our previously described congenital glaucoma cohort^6^,^7^. The selected cases manifested PCG diagnosed before the fourth month of life and most of them had no family history of glaucoma or consanguinity. The initial aim of the study was to find rare, coding and recessive variants located in genes impaired in at least 10% of the patients, i.e., three or more patients. We did not find any variant that fit this model (Fig. 1A), indicating the existence of *locus* heterogeneity among these subjects. Next, as an alternative strategy, we focused our search on private variants (i.e., those present in a single patient) of genes that control ocular development throughout regulation of gene expression, which can be identified by the presence of nucleic acid-binding domains sequences. Based on this approach, among an average of 60 variants located in well-supported transcripts per subject, we selected as candidates two *GPATCH3 (G-PATCH DOMAIN-CONTAINING PROTEIN 3*) variants present in patient PCG-99 (Fig. 1B). These nucleotide changes (c.701A > G, p.Asn234Ser and c.1424 G > A, p.Gly475Glu) were present in the compound heterozygous state, were confirmed by Sanger sequencing (Supplementary Fig. 1), cosegregated with the disease phenotype in this family according to an autosomal recessive inheritance (Fig. 1C,D), and were not detected in a group of 190 (380 chromosomes) ethnically matched control subjects. In addition, these two mutations affected highly conserved amino acid residues (Phylop score > 0.98, values range between 0 and 1)^16^ and were predicted to be probably or possibly damaging according to PolyPhen scores (Table 1). *GPATCH3* is a seven-exon gene of unknown function, that encodes a protein predicted to contain the G-patch domain, which has been described to be involved in protein-nucleic acid interactions and is present in both RNA- and DNA-binding proteins^17^.

### Additional *GPATCH3* variants in congenital glaucoma

**Figure 2:**
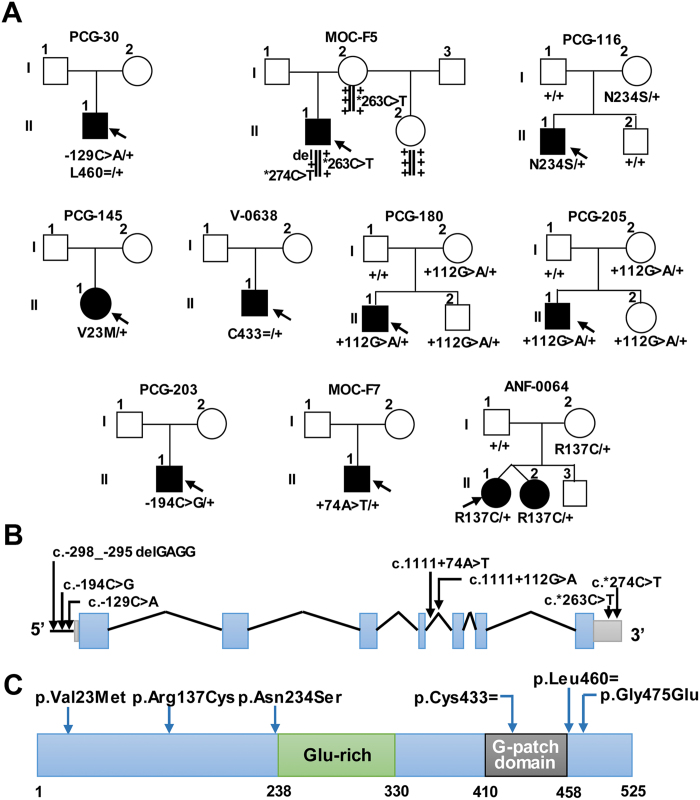
Pedigree and segregation analysis of *GPATCH3* variants in families with congenital glaucoma. (**A**) Segregation and pedigrees of the families. Black symbols indicate congenital glaucoma. The arrows in the pedigree show the index case. +: wild-type allele. The vertical lines below the symbols represent chromosomes. −129C > A: c.−129C > A. L460 = : p.Leu460 = . del: c.−298_−295delGAGG. *263 C > T: c.*263C > T. *274C > T: c.*274C > T. N234S: p.Asn234Ser. V23M: p.Val23Met. C433 = : p.Cys433 = . + 112G > A: c.1111 + 112G > A. −194C > G: c.−194C > G. + 74A > T: c.1111 + 74A > T. R137C: p.Arg137Cys. (**B**) Localization of non-coding *GPATCH3* mutations identified in this study. Structure of the gene indicating the exons (blue) and the position of the promoter (horizontal line), intronic (oblique lines), 5′- and 3′-UTR variants (green). (**C**) Structure of the polypeptide chain indicating the position of the coding variants and the predicted G-patch domain and Glu-rich region reported in the UniProt database.

To assess the presence of rare *GPATCH3* variants in congenital glaucoma patients, we screened this gene by Sanger sequencing in a cohort of 130 unrelated congenital glaucoma cases with no known gene mutations and in a second cohort composed of 40 secondary congenital glaucoma cases associated with ocular malformations such as microphthalmia or anterior segment dysgenesis. We identified 10 (5.8%) patients who carried a total of twelve rare *GPATCH3* variants in the heterozygous state (Table 1 and Fig. 2A), located both in coding and non-coding regions of the gene (Fig. 2B and C). Almost all of these patients manifested bilateral glaucoma with early diagnosis (Supplementary Table 4). All of these variants were absent in our ethnically matched control group and four of them were not present in the databases used in this study as references (Table 1). Interestingly, two of these probands were compound heterozygotes for these rare variants. The first of them (PCG-30) carried nucleotide changes located in the promoter (c.−129C > A) and in the coding region (p.Leu460 = ) (Fig. 2A), which were predicted to alter a putative transcription factor binding site (AML-1, Supplementary Fig. 2) and to affect splicing by disrupting a putative exonic splicing enhancer (Table 1), respectively. Progenitors’ DNA samples were not available for segregation analysis. The second proband (MOC-F5), diagnosed with secondary glaucoma and iridocorneal endothelial syndrome, presented three rare non-coding *GPATCH3* variants (c.-298_−295delGAGG, c.*263C > T and c.*274C > T) (Fig. 2A) with frequencies equal to or less than 1% (Table 1). Segregation analysis in this family showed that variants c.-298_−295delGAGG and c.*274C > T were inherited on the same chromosome as an individual haplotype (Fig. 2A). Moreover, the segregation was compatible with an autosomal recessive inheritance and the three variants were absent in our control group of 190 ethnically matched subjects. The c.−298_−295delGAGG variant was located in the core promoter region (Fig. 2B) and affected the nucleotide sequence of putative binding sites predicted for two different transcription factors (E74A and RXR-VDR) (Supplementary Fig. 2). The c.*263C > T and c.*274C > T variants were localized in the 3′UTR sequence (Fig. 2B), affecting inferred seed target sequences for various miRNAs (Supplementary Fig. 2). Among the remaining five heterozygous coding variants, we found three non-synonymous nucleotide changes (including the previously identified p.Asn234Ser in patient PCG-99). All affected amino acids were conserved in mammals, except p.Asn234 and p.Leu460 which were conserved from mammals to fishes (Supplementary Fig. 2), and p.Val23Met and p.Arg137Cys were predicted to be damaging according to PolyPhen and SIFT scores, respectively (Table 1). The synonymous p.Cys433 = , p.Leu460 = and intronic c.1111 + 112G > A variants were inferred to disrupt exon splicing by different mechanisms (Table 1). No functional defect was inferred for the second intronic variant (c.1111 + 74A > T) with the programs used. Overall, these results show two additional cases with a potential recessive inheritance and an unexpectedly high frequency of heterozygous carriers of rare variants in these cohorts, indicating a putative role for the *GPATCH3* mutations as causative, modifier or susceptibility factors in the disease.

### *GPATCH3* expression in human ocular tissues

**Figure 3:**
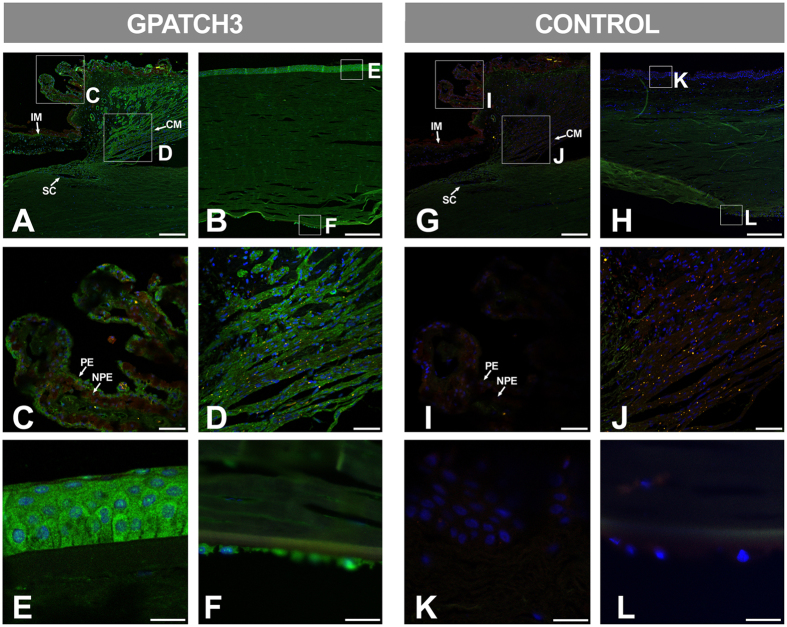
Fluorescent immunohistological detection of GPATCH3 in anterior segment tissues of a human eye. (**A**–**L**) Fluorescent immunohistochemistry of histological sections (10 μm) of a human eye from a 45-year-old Caucasian female donor (cadaver) with no reported ocular pathology. Samples were incubated with either rabbit anti-GPATCH3 primary antibody and Cy2 donkey anti-rabbit secondary antibody (**A**–**F**) or only secondary antibody as a negative control (**G**–**L**). Confocal wide-field micrographs of iridocorneal angle (**A** and **G**) or cornea (**B** and **H**) and detailed images of ciliary processes (**C** and **I**), ciliary muscle (**D** and **J**), corneal epithelium (**E** and **K**) and endothelium (**F** and **L**) are shown. Scale bars indicate 200 μm in panels A, B, G and H, 50 μm in panels C, G, I and J and 20 μm in panels E, F, K and L. The green channel corresponds to GPATCH3, the red channel to tissue autofluorescence and the blue channel to DAPI nuclear staining. IM: Iris muscle. CM: Ciliary muscle. SC: Schlemm channel. PE: Pigmented epithelium. NPE: Non-pigmented epithelium.

Tissue expression of *GPATCH3* has not been previously reported. Therefore, we determined the presence of the protein in available samples of human ocular and non-ocular tissues by Western blot. The analysis revealed a principal high-molecular weight band and secondary bands of more than 120 kDa, and approximately 85 kDa and 55 kDa in all tissues studied (Supplementary Fig. 3). According to the theoretical molecular weight of this 525-amino acid-long protein (59.3 kDa), the lower band could correspond to the monomer and the rest to different protein aggregates. The 85 kDa and 55 KDa bands were also observed in a control sample consisting of the recombinant protein transiently expressed in HEK-293T cells, supporting the specificity of the identified bands. Next, we investigated by immunohistochemistry the distribution of this protein in human ocular tissues relevant to glaucoma obtained from a 45-year-old Caucasian female donor (cadaver) with no reported ocular pathology. We observed specific GPATCH3 labeling in non-pigmented ciliary epithelium (NPE) (Fig. 3A and C) and anterior segment structures, particularly in the ciliary muscle (Fig. 3A and D). In addition, both the corneal epithelium and endothelium were also labelled (Fig. 3B,E and F). The main GPATCH3 signal was detected in the cytoplasm of the corneal epithelium, but it was also observed in sub-nuclear structures (Fig. 3E). These signals were not detected in the corresponding negative controls (Fig. 3G–L).

### Subcellular localization of wild-type *GPATCH3* and its coding variants

To study the wild-type protein and the identified coding variants *in vitro*, we used a commercial cDNA encoding this protein (OriGene), subcloned into the pcDNA 3.1(−) myc-his vector (Supplementary Methods section). The two coding variants identified in patient PCG-99 were obtained by site-directed mutagenesis and cloned in the same vector, as indicated in the Methods section. Fluorescence immunocytochemistry of recombinant wild-type GPATCH3 transiently expressed in HEK-293T cells showed that the protein localized mainly in the nucleus, with a pattern similar to that of the transcription factor FOXC2, which is known to be involved in ocular development^18^, and was used as a positive control (Supplementary Fig. 4). This result also indicated the role of GPATCH3 as a putative nucleic acid binding protein. The p.Asn234Ser and p.Gly475Glu variants identified in congenital glaucoma patients showed similar nuclear localization (Supplementary Fig. 4), revealing that these two amino acid changes do not alter subcellular localization in cells in culture.

### Increased transactivation of the *CXCR4* promoter by some *GPATCH3* variants

**Figure 4:**
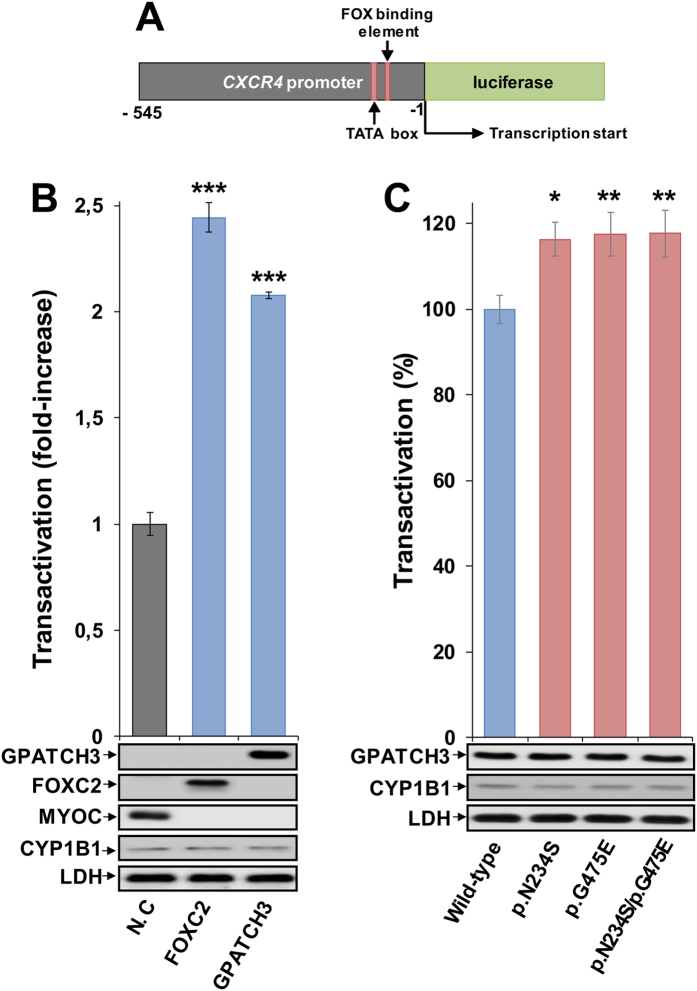
Transactivation of the *CXCR4* promoter by GPATCH3 and increased activity of variants p.Asn234Ser and p.Gly475Glu. (**A**) Scheme of the cDNA construct containing a FOX-binding element and the TATA box sequence present in the CXCR4 promoter fused to the luciferase coding region. The cDNA was cloned into the PGL3 basic vector. This construct was used as a reporter of transcriptional activity in co-transfection assays with cDNAs encoding the transcription factor FOXC2 (positive control), the extracellular protein myocilin (MYOC, negative control), the different GPATCH3 variants and the intracellular protein CYP1B1 (transfection efficiency control). The numbers below the scheme correspond to nucleotide positions. (**B**) cDNA constructs encoding MYOC, FOXC2 and GPATCH3 were transiently co-expressed with the reporter luciferase cDNA in HEK-293T cells. The transcriptional activity, expressed as fold increase of the luciferase activity of MYOC (negative control, N.C), was measured as indicated in the Methods section. (**C**) cDNA constructs encoding the different variants of GPATCH3 were transiently co-expressed with the reporter luciferase cDNA in HEK-293T cells. The transcriptional activity, expressed percentage of the luciferase activity of wild-type GPATCH3, was measured as indicated in the Methods section. The protein levels present in HEK-293T cells 24 h after transfection were determined by western immunoblot using a monoclonal anti-myc antibody. Each lane contained 15 μg of total protein obtained from the cell lysates. Transfection efficiency (CYP1B1) was assessed via western immunoblot using a monoclonal anti-myc antibody. The sample loading control, endogenous LDH, was also detected via immunoblot using an anti-LDH antibody. Values are expressed as mean ± SEM of at least three independent experiments carried out in triplicate. Asterisks indicate statistical significance as compared to the control: p < 0.02 (*); p < 0.006 (**); p < 0.001 (***). Statistical significance was calculated by Student’s t-test.

The presence of the G-patch nucleic acid-binding domain in the polypeptide chain of GPATCH3 and its nuclear localization, similar to that observed for the transcription factor FOXC2, supported the hypothesis that GPATCH3 might regulate gene expression. To investigate this, we used a luciferase assay previously set up in our laboratory to measure FOXC2 transactivation using the *CXCR4* promoter^19^. Transient co-expression in HEK-293T cells of a cDNA construct encoding luciferase coupled to the *CXCR4* proximal promoter region, with cDNA constructs encoding FOXC2 (positive control) or GPATCH3, showed a luciferase activity 2.4-fold and 2.0-fold higher than that of the negative control, respectively, showing a positive effect of GPATCH3 on transactivation, close to the level of the transcription factor FOXC2 (Fig. 4B). This result indicated that GPATCH3 play a role as a gene expression regulator. Next, we evaluated whether the two *GPATCH3* variants identified in patients PCG-99 (p.Asn234Ser and p.Gly475Glu) affected *CXCR4* transactivation in this assay. We observed an approximately 17% increase in transactivation activity compared with the wild-type protein (Fig. 4C), indicating that the amino acid changes result in hypermorphic variants, and revealing a functional alteration induced by the two amino acid changes. Consistent with these results, a simulation of the heterozygous genotype found in patient PCG-99, carried out by transient co-expression of both variants in the cell lines, also showed a 17% increase in transcriptional activity. Western blot analyses of the GPATCH3, CYP1B1 and LDH proteins showed no significant differences in GPATCH3 protein expression, transfection efficiency or sample loading in any of these assays (Fig. 4).

### Expression of Gpatch3 in zebrafish dermis, skeletal muscles, head cartilages, corneal endothelium and periocular mesenchymal-like cells

**Figure 5:**
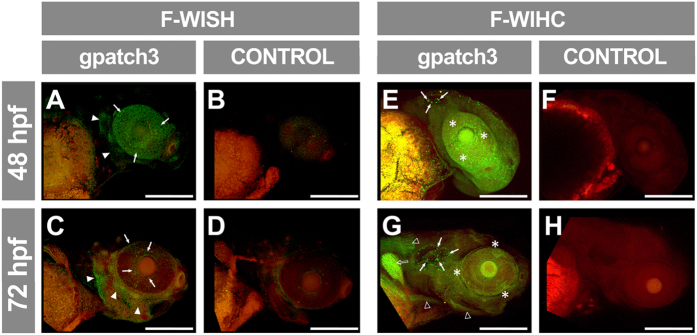
Analysis of *gpatch3* expression in ocular and non-ocular embryonic tissues during early zebrafish development. (**A**–**D**) Expression analysis by fluorescent-whole mount *in situ* hybridization (F-WISH) of *gpatch3* in zebrafish embryos at 48 (**A** and **B**) or 72 (**C** and **D**) hpf with Alexa Fluor-488 labelled *gpatch3* antisense RNA probes (**A** and **C**) or sense RNA probes as a control (**B** and **D**). The green channel corresponds to *gpatch3* expression and the red channel to tissue autofluorescence. The arrows and arrowheads indicate *gpatch3* expression in the developing anterior segment and head dermis, respectively. Scale bars represent 200 μm. (**E**–**H**) Immunodetection by fluorescent-whole mount immunohistochemistry (F-WIHC) of Gpatch3 in zebrafish embryos at 48 (**E** and **F**) or 72 hpf (**G** and **H**) using rabbit anti-GPATCH3 primary antibody and Cy2 donkey anti-rabbit secondary antibody (**E** and **G**) or only secondary antibody (**F** and **H**) as a negative control. The green channel corresponds to Gpatch3 expression and the red channel to tissue autofluorescence. Arrows, empty arrowheads, empty arrow and asterisks indicate Gpatch3 expression in the otic vesicle, muscles, pectoral fin and developing anterior segment, respectively. Scale bars represent 200 μm.

**Figure 6:**
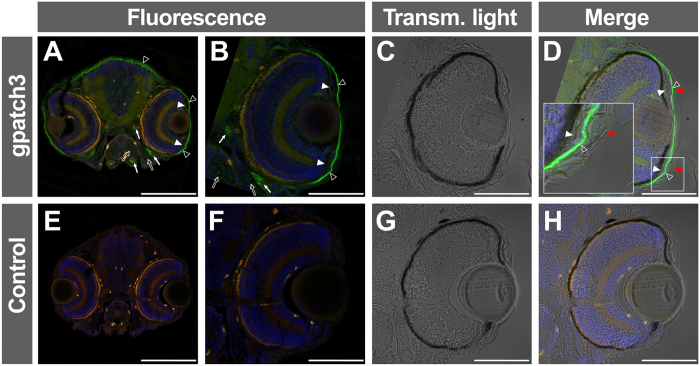
Immunohistological detection of Gpatch3 in transverse head sections of zebrafish embryos. (**A**–**D**) Expression of *gpatch3* was analyzed in 10 μm cryostat sections of fixed zebrafish embryos at 96 hpf by fluorescent immunohistochemistry with a rabbit anti-GPATCH3 primary antibody and a Cy2 donkey anti-rabbit secondary antibody. The green channel corresponds to Gpatch3, the red channel to tissue autofluorescence and the blue channel to DAPI nuclear staining. The arrows indicate mandibular and periocular muscles, the empty arrows indicate jaw cartilages, the white arrowheads indicate periocular mesenchyme, the empty arrowheads indicate corneal endothelium and dermis and the red arrowheads show the corneal epithelium. (**C** and **G**) Transmitted light microscopy of panels B and F, respectively. (**E–H**) Embryos hybridized with only secondary antibody were used as a negative control. Scale bars represent 200 μm in panels A and E and 100 μm in panels (**B**–**D** and **F**–**H**).

Further functional characterization of *GPATCH3* and evaluation of its role in congenital glaucoma was carried out in zebrafish because this animal model has been used for the study of glaucoma^20^,^21^ and it is very efficient for analyzing ocular and general development. A homology search identified a single *GPATCH3* orthologue located on chromosome 16 of the *D. rerio* genome, with well conserved intron-exon organization (Supplementary Fig. 5) and high amino acid sequence similarity (49.3%) (Supplementary Fig. 5). The main difference between the human and zebrafish genes consisted of an 1112 nucleotide-long insertion in exon 2 of the *D. rerio* gene that encodes an amino acid sequence characterized by the presence of 20 Pro-Thr/Ser repetitions regularly distributed every 7–9 amino acid residues (Supplementary Fig. 5). *Gpatch3* expression was assessed in zebrafish embryos by fluorescent-whole mount *in situ* hybridization (FWISH) and fluorescent-whole mount immunohistochemistry (FWIHC) at 48 and 72 hours post fecundation (hpf). At 48 hpf, FWISH revealed an apparently superficial signal with mosaic pattern in the eye and some areas of the head (Fig. 5A, arrowheads). At 72 hpf the intensity of the specific signal decreased particularly in the anterior, posterior and ventral eye quadrants and persisted in the dorsal part of the eye, periocular region and precursor area of the anterior segment angle (Fig. 5C, arrows). We observed a parallel increased signal in the rest of the head (Fig. 5C, arrowheads). Detection of the endogenous Gpatch3 protein by FWIHC supported these results, and it revealed a dot-like staining of the otic vesicle (Fig. 5E and G, arrows) and labelling of the muscles of the head, neck and trunk (Fig. 5G, empty arrowheads), as well as of pectoral fins (Fig. 5G, empty arrow). In the eye, the signal was irregularly distributed and was more intense in periocular tissues (Fig. 5E and G, asterisks). Fluorescent immunohistochemical analysis of frozen sections by confocal microscopy confirmed that the Gpatch3 signal was superficially distributed, with intense labeling of the corneal endothelium and dermis (Fig. 6A and B, empty arrowheads). Gpatch3 was also located in jaw muscles and in other muscles of the head (Fig. 6A and B, arrows). Weak signals in the jaw cartilages were also observed (Fig. 6A and B, empty arrows). Interestingly, cells in the angle of the anterior segment also showed weak Gpatch3 signals (Fig. 6B, white arrowheads). Light and fluorescence image overlapping revealed that the labeled cells (Fig. 6D, empty arrowheads) were situated below the incipient epidermis and corneal epithelium (Fig. 6D, red arrowheads).

### *GPATCH3* is involved in ocular and craniofacial development in zebrafish

**Figure 7:**
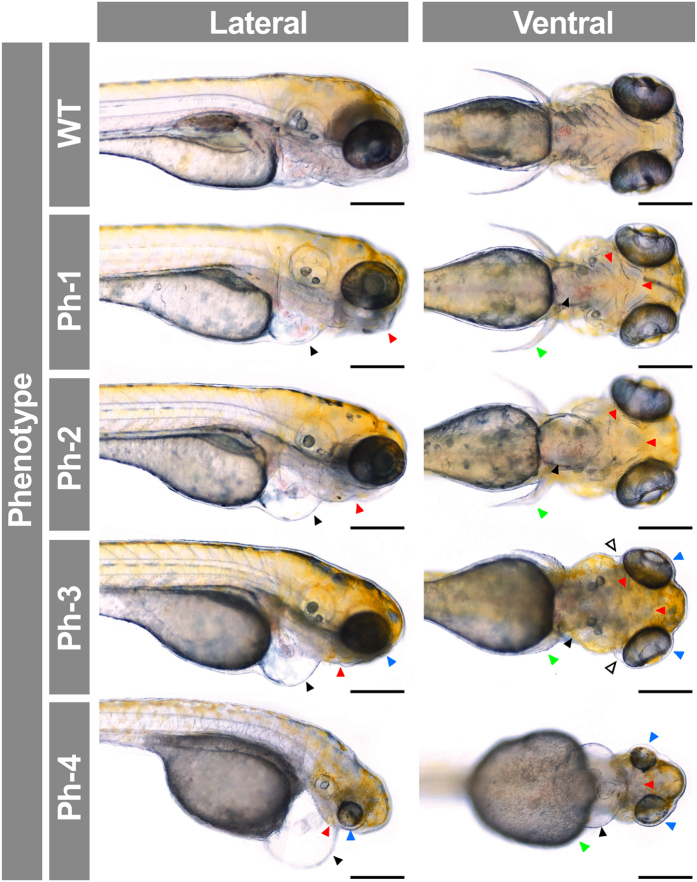
Analysis of *gpatch3* knock-down-associated phenotypes in zebrafish morphant embryos. *Gpatch3*^*ATG*^ MO-injected zebrafish embryos were analyzed at 96 hpf, and four phenotypes (Ph-1-4) with increasing severity were described. Lateral and ventral brightfield micrographs show altered phenotypes including microphthalmia (blue arrowheads), mouth and branchial arches (red arrowheads) and pectoral fins (green arrowheads) maldevelopment and pericardial (black arrowheads) and periocular (empty arrowheads) edemas. Scale bars represent 200 μm.

**Figure 8:**
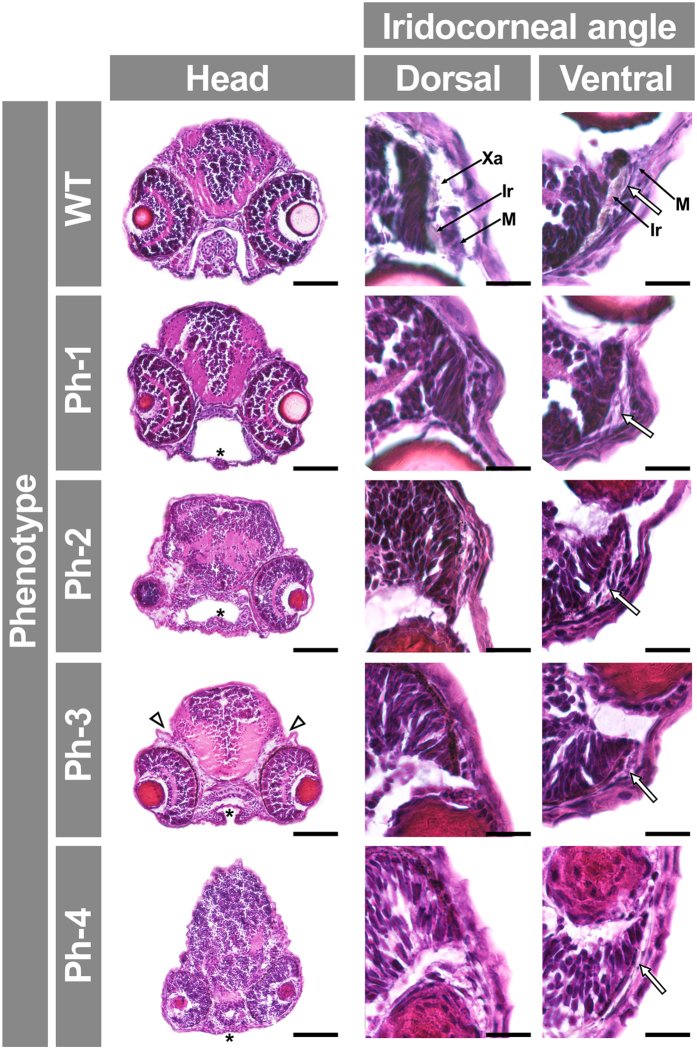
Analysis of *gpatch3* knock-down-associated phenotypes in histological transverse sections of zebrafish *gpatch3* morphants. *Gpatch3*^*ATG*^ MO-injected zebrafish embryos were analyzed at 96 hpf and four phenotypes (Ph-1-4) with increasing severity were described. The 100X and 400X brightfield micrographs of 10 μm cryostat transverse head sections stained with hematoxilin and eosin show altered phenotypes including microphthalmia, mandibular maldevelopment (asterisks), enlarged intracranial and periocular spaces (empty arrowheads) and iridocorneal angle defects (empty arrows). Scale bars represent 100 μm in head section panels and 20 μm in iridocorneal angle magnification panels. Xa: xantophores. Ir: iridophores. M: periocular mesenchyme.

To assess whether *gpatch3* is involved in ocular development we transiently suppressed its expression via three non-overlapping, translation- (*gpatch3*^*ATG*^) or splice-blocking morpholinos (MOs) targeting exons 1 (*gpatch3*^*ex1/2*^) and 3 (*gpatch3*^*ex2/3*^) (Supplementary Fig. 5). Gene suppression in embryos injected with 4.0 ng of either of the three MOs demonstrated similar gross phenotypes characterized by variable degrees of abnormal ocular, fin, head and jaw morphology (Supplementary Fig. 6). The *gpatch3*^*ATG*^ MO was selected for further studies. To determine the optimal MO amount, embryos were injected with 2.0, 4.0 or 8.0 ng of *gpatch3*^*ATG*^. Two nanograms of MO were used in further experiments because this amount yielded a higher proportion of mild phenotypes and the lower lethality (data not shown). An initial morphological evaluation of morphants and control embryos was carried out using 200 embryos in each experiment. At 24 hpf 11% of control-MO injected embryos showed aberrant morphology, characterized by curved body and lethality and were considered non-specific phenotypes. The proportion of lethal phenotypes increased to 53% in *gpatch3*^*ATG*^-injected embryos, suggesting a *gpatch3* knock-down specific effect. Surviving embryos with altered ocular and cranial morphology (35%) were observed from 24 hpf and became severely abnormal at 96 hpf. Most of these embryos survived until 7 days post fertilization. *Gpatch3* morphants were classified into four abnormal phenotypic classes according to increasing degree of microphthalmia, microcephaly, pericardial edema and maldevelopment of cartilages present in pectoral fins, jaws and branchial arches (Fig. 7). These features were not observed in either control MO-injected larvae or uninjected embryos (data not shown), indicating that they were specific. At 96 hpf 90% of control morpholino-injected animals showed a normal appearance but only 12% of *gpatch3*^*ATG*^-injected embryos (Supplementary Fig. 7) and almost 100% of uninjected embryos (data not shown) exhibited an unaffected phenotype. Western blot analysis of protein extracts from the four types of *gpatch3* morphants revealed a significant 60–90% reduction in Gpatch3 protein levels associated with the severity of the abnormal phenotypes, indicating the correct targeting and efficiency of the MO treatment (Supplementary Fig. 8). Next, we focused on histological alterations of tissues relevant to glaucoma. Hematoxylin and eosin-stained eye sections of *gpatch3* morphants at 96-hpf revealed defects in ocular development (Fig. 8). The main alteration was observed in the anterior segment angle and in the periocular tissue. The dorsal angle showed a remarkable and MO dose-dependent hypoplasia characterized by decreased silver and foamy cells, which correspond to iridophores and xantophores, respectively (Fig. 8), along with an apparent accumulation of undifferentiated mesenchymal-like cells in the anterior segment angle (Fig. 8). Development of the ventral angle was also delayed and hypoplastic (Fig. 8, right-hand panels, see empty arrows). In addition, we detected increased periocular spaces (Fig. 8, Ph-3, head, empty arrowhead), which was also observed *in vivo* (Fig. 7, Ph-3, ventral view, empty arrowhead) and may reflect the existence of periocular edema. The histological study also revealed that the pharyngeal cartilages were underdeveloped and even absent in the most severe phenotypes (Fig. 8, asterisks). Alcian blue staining of morphants confirmed the abnormal development of the chondrocranial and pharyngeal cartilages, including shortening of the ethmoid plate, progressive dysplasia of Meckel’s and palatoquadrate cartilages, and progressive disappearance of ceratohyal and ceratobranchyals cartilages (Supplementary Fig. 9). Similar abnormalities were observed by confocal microscopy analysis of morphants obtained using the fluorescent transgenic reporter Tg(*sox10*:eGFP), which present endogenous fluorescence in neural crest-derived cell populations^22^ (Supplementary Fig. 10). Interestingly, some of these phenotypic features resembled those observed in *pitx2*-knockdown zebrafish embryos (e.g., increased periocular spaces and jaw and pharyngeal arch disruption)^23^.

**Figure 9:**
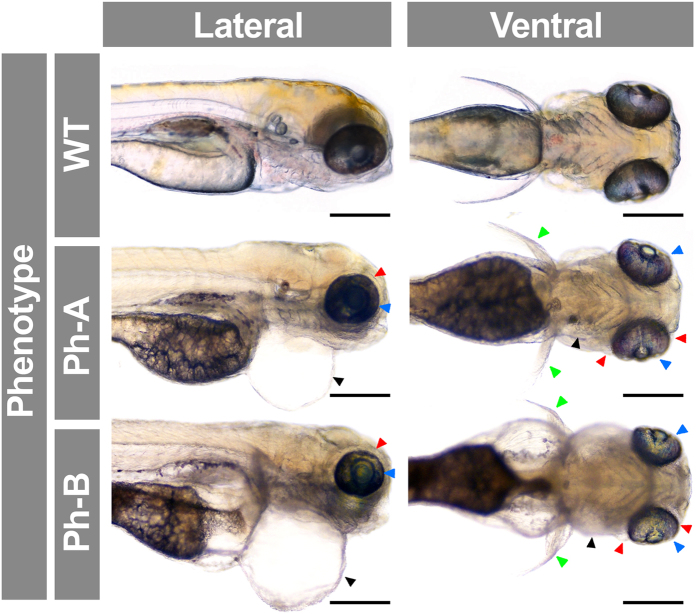
Analysis of *gpatch3* overexpression-associated phenotypes in zebrafish embryos. *Gpatch3* mRNA microinjected zebrafish embryos were analyzed at 96 hpf and two phenotypes (Ph-A- and Ph-B) with increasing severity were described. Lateral and ventral brightfield micrographs show altered phenotypes including microphthalmia (blue arrowheads), pectoral fins maldevelopment (green arrowheads) and periocular (red arrowheads) and pericardial (black arrowheads) edemas. Scale bars represent 200 μm.

**Figure 10:**
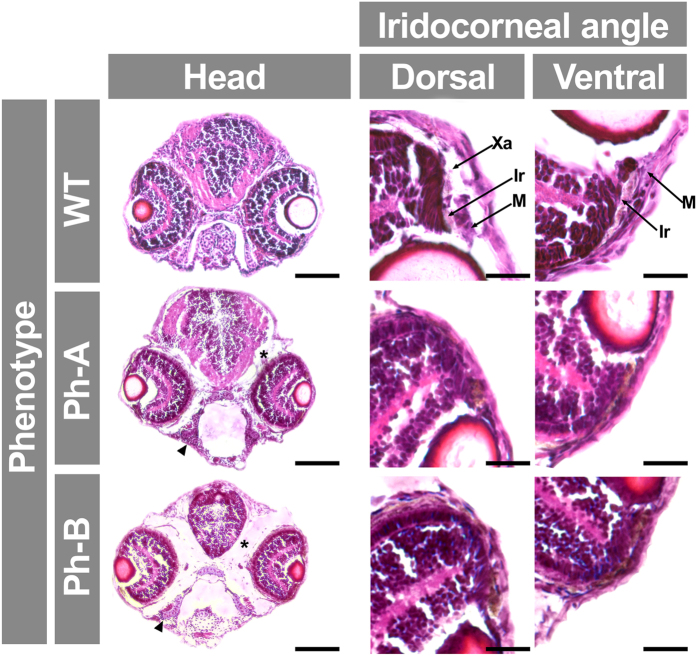
Analysis of *gpatch3* overexpression-associated phenotypes in histological transverse sections of zebrafish. *Gpatch3* mRNA microinjected zebrafish embryos were analyzed at 96 hpf and two phenotypes (Ph-A and Ph-B) with increasing severity were described. Brightfield micrographs of 10 μm cryostat transverse head sections stained with hematoxilin and eosin show altered phenotypes including microphthalmia, iridocorneal angle defects, periocular and intracraneal edemas (asterisks) and underdevelopment of pharyngeal cartilages (arrowhead). Scale bars represent 100 μm in head section panels and 20 μm in iridocorneal angle magnification panels. Xa: xantophores. Ir: iridophores. M: periocular mesenchyme.

To determine the specificity of the MO effect, we evaluated the reversal of MO phenotypes using mRNA rescue. The *gpatch3*^*ATG*^ MO (2.0 ng) was co-injected with zebrafish *gpatch3* mRNA (0.4 ng) in 1–2 cell embryos; this resulted in a reduced proportion of lethal phenotypes (from 54% to 12%) and parallel increase in wild-type-like phenotypes (from 11% to 51%), indicating that the rescue was complete in approximately 40% of the embryos, and partial in the rest (Supplementary Fig. 7). In an effort to assess the effect of *gpatch3* hypermorphic variants *in vivo,* we transiently increased the amount of this protein in zebrafish embryos by microinjecting *gpatch3* mRNA in the yolk of 1–2 cell embryos. Embryos were microinjected with 0.4 ng of *in vitro*-transcribed *gpatch3* mRNA. Approximately 10% of the microinjected embryos showed normal phenotypes and 45% exhibited lethality (Supplementary Fig. 7). The remaining phenotypes were classified into two types (Ph-A and Ph-B) characterized by increasing gross abnormalities consisting mainly of variable degrees of pericardial, yolk sac and periocular edema and microphthalmia (Fig. 9). Interestingly, and in contrast with knockdown embryos, the pharyngeal arch cartilages were less affected. A detailed histological analysis of these phenotypes revealed increased spaces around the eyes and brain filled with an amorphous substance (Fig. 10, asterisk). The extent of these empty spaces correlated with reduced eye and brain size, which may be the result of edema. Variable and progressive underdevelopment of the pharyngeal cartilages (e.g., basihyal) was also observed. Interestingly, a remarkable anterior chamber angle hypoplasia was present, with a decreased number of iridophores and particularly of foamy cells (xantophores) (Fig. 10). These results show that increased Gpatch3 activity also disrupts early embryo development, inducing some glaucoma-related ocular abnormalities.

## Discussion

We conducted a WES study in 26 cases with severe PCG to identify candidate genes present in more than one patient, interrogating the exome for candidate rare variants present in three or more patients and inherited according to a monogenic recessive model. This initial searching strategy did not reveal any candidate genes, in accordance with a recent report, in which no candidate genes were identified by filtering for novel recessive variants in a single gene in 21 affected individuals^24^. These data support the existence of high genetic heterogeneity of this disease in different populations, which will likely complicate the identification of new congenital glaucoma-causing genes. Given the possibility of high *locus* heterogeneity among this group of patients and based on the idea that PCG results from maldevelopment of the aqueous outflow system, we focused our search on private variants of genes potentially involved in ocular development, many of which regulate gene expression. This strategy identified candidate disease-causing *GPATCH3* variants in only one patient (PCG-99). *GPATCH3* maps to chromosome 1 and encodes a protein of unknown function, so named because of the presence of the G-patch domain. This gene was considered a good candidate and a putative gene expression regulator because the G-patch domain has been proposed to participate in protein-nucleic acid interactions^25^ and is present in both RNA- and DNA-binding proteins, including the splicing factor SPF-45^25^ and the transcription repressor ZIP (zinc finger and G-patch domain-containing protein)^26^. The G-patch domain is approximately 48 amino acids-long and contains six highly conserved glycine residues^25^. Further screening of two cohorts of primary congenital glaucoma and secondary congenital glaucoma associated with ocular malformations, including microphthalmia and anterior segment dysgenesis, identified another patient with new candidate variants in this gene. In these two patients, who represent approximately 1% of the total number of cases included in the study, the variants were inherited according to an autosomal recessive pattern. In addition, approximately 5% of the patients in these cohorts carried *GPATCH3* rare variants in the heterozygous state. Segregation analysis of these variants failed to reveal a monogenic inheritance, but their elevated frequency among patients and the predicted functional impact for most of them suggest that they are disease-causing variants that may be involved in non-monogenic congenital glaucoma in accordance with our previous findings^6^,^7^,^12^,^19^. Also in accordance with this hypothesis, a recent study reported that 10 of 189 unrelated PCG families carried heterozygous rare variants in the *TEK* gene with dominant transmission and highly variable expressivity, which was explained by stochastic developmental events or oligogenic/digenic inheritance^24^. In any event, non-monogenic glaucoma inheritance may result from genetic disruption of the complex network of gene interactions that regulate ocular anterior segment development. The pathogenicity of the identified variants was initially supported by their low frequency in the general population and in controls, their evolutionary conservation and the predicted effect on protein function. One of the SNVs identified in patient PCG-99 (p.Asn234Ser) has been reported in the homozygous state in two subjects from the non-Finnish European sample contained in the ExAC database (http://exac.broadinstitute.org/). The finding can be explained if we take into consideration the possibility of incomplete penetrance, a phenomenon known to be associated with variants which, like this one, are not completely detrimental to protein function^27^. On the other hand, it cannot be definitively ruled out that the two homozygotes do not present any glaucoma-related feature, since large population databases do not contain extensive information regarding any possible associated phenotype^28^. All variants identified in glaucoma patients, except c.111 + 74A > T, were bioinformatically predicted to dysregulate *GPATCH3* function not only at the protein level but also at the level of transcription or translation, affecting the dosage of the gene product, suggesting that they are disease-causing-variants. In addition, the functional analyses carried out both *in vitro* and *in vivo* using zebrafish as an animal model also supported the involvement of this gene disruption in PCG. Our results showed that the recombinant protein expressed in HEK-293T cells behaves as a nuclear protein, with a pattern similar to that of the transcription factor FOXC2. Moreover, i*n vitro* transactivation assays revealed that the wild-type GPATCH3 protein activated the *CXCR4* core promoter, further indicating its role in transcriptional regulation. The activation of this promoter can be due to direct binding of the GPATCH3 protein to regulatory DNA sequences or to an indirect mechanism involving interactions with other transcriptional regulators. Further studies are required to elucidate this point. On the other hand, the two coding mutations identified in patient PCG-99 showed that they are hypermorphic variants with moderate increased activity. These data are particularly interesting because they indicate that *GPATCH3* may regulate the expression of *CXCR4*, a gene that encodes a chemokine receptor involved in neural crest cell migration^29^,^30^ and eye field organization in the early embryo^31^. Therefore, it can be hypothesized that *GPATCH3* could regulate ocular and craniofacial development by controlling neural crest cell migration. At this time, the precise mechanism involved in the effect of GPATCH3 on *in vitro* gene expression remains uncharacterized and requires further study. The moderate increased activity associated with the identified *GPATCH3* mutations may explain why the disease phenotype in our patients is non-syndromic. Therefore, we can postulate the existence of *GPATCH3* dose-dependent phenotypes. In accordance with this idea, it is known that normal anterior segment development depends on precise doses and activity levels of certain transcription factors such as *FOXC1, FOXC2* and *PITX2*^32^. Activity-dependent phenotypes have also been proposed for *CYP1B1*^6^,^7^.

Western blot and immunohistochemistry revealed consistent expression of GPATCH3 in glaucoma-related adult human ocular tissues. In the adult human corneal epithelium the protein was mainly detected in the cytoplasm and also in the nuclei. Based on this data we hypothesize the existence in these adult cells of a population of GPATCH3 molecules that are synthesized in the cytoplasm and travel to the nucleus in response to specific signals. In an effort to assess the involvement of *GPATCH3* in ocular development and glaucoma, we studied the expression and function of the zebrafish orthologue in early embryos. In accordance with the results obtained in human tissues, both FWISH and FWIHC revealed the presence of the Gpatch3 protein in the dermis and skeletal muscles of the head and jaw of early zebrafish embryos (48 and 72 hpf). Remarkably, it was also detected in the corneal endothelium and other periocular mesenchymal cells, including cells of the ciliary and canalicular zones, as well. The expression pattern of *gpatch3* in some of these embryonic ocular structures (e.g. cornea, dermis and periocular mesenchyme) were similar to those observed for *pitx2*^33^ and overlapped with the presence of *foxc1* in the periocular mesenchyme and cornea^34^. At this stage of development, the periocular mesenchyme, a subpopulation of the cranial neural crest cells, surrounds the emerging eye giving rise to the specialized structures of the anterior segment of the eye that are responsible for aqueous humor dynamics^35^,^36^. The functional disruption of *gpatch3* by three different MOs resulted in a number of similar zebrafish developmental abnormalities including ocular malformations and craniofacial defects in structures derived from the pharyngeal arches (e.g., jaw cartilages). Significant off-target effects were unlikely as we observed similar abnormal phenotypes with the three different MOs assayed. Microinjection of similar amounts of a standard negative control did not show apparent malformations, indicating the absence of non-specific toxic effects in morphants. In addition, the mRNA rescue assays and the reduction in Gpatch3 protein levels associated with the severity of the abnormal phenotypes supported that the MO effects were specific. The expression pattern of this gene and the affected tissues point to possible defects in differentiation and migration of neural-crest derived cells, as previously mentioned. The morphants presented a lack of differentiation and dysgenesis of anterior segment structures, mimicking important features of congenital glaucoma phenotypes. Moreover, evaluation of the effect of hypermorphic variants by temporary mRNA overexpression in zebrafish embryos resulted in yolk sac, pericardial and periocular edemas and alteration of jaw cartilages. Pericardial edema was also associated with reduced *gpatch3* expression and is a phenotypic feature shared with *foxc1*^34^- or *pitx2*^23^-deficient zebrafish. Another remarkable connection with congenital glaucoma, was the presence of iridocorneal angle hypoplasia, accompanied by a decreased number of neural crest-derived cells such as iridophores and xantophores. Overall, these phenotypes recapitulate some of the ocular and craniofacial features observed in *pitx2*^23^,^33^- or *foxc1*^34^-deficient zebrafish. Altogether, these data indicate that *gpatch3* could participate in the intricate gene network and molecular signaling that control eye and jaw morphogenesis, suggesting a functional relationship with *pitx2* and *foxc1* and providing evidence for a role of *GPATCH3* in the complex genetic etiology of congenital glaucoma.

## Methods

### Subjects

Twenty-six severe PCG probands were selected from a cohort composed of 150 unrelated families affected by PCG with no *CYP1B1* or *MYOC* mutations. The Ethics Committee for Human Research of the Hospital Clínico San Carlos approved the study and informed consent procedures (approval number 13/388-E), in accordance with the tenets of the Declaration of Helsinki. Informed written consent was obtained from all of the participants and was recorded by the staff involved in the study. The clinical features of the majority of these patients have been reported^6^,^7^. The severe congenital glaucoma phenotype was defined as glaucoma diagnosed before the 4^th^ month of life.

All subjects were clinically evaluated by glaucoma specialists. The ophthalmic examination included slit lamp biomicroscopy, gonioscopy, biometry, intraocular pressure (IOP) measurement and ophthalmoscopy. The PCG clinical diagnosis was performed as previously described^6^. Control individuals were recruited from among those who attended the clinic for conditions other than glaucoma, including cataracts, floaters, refractive errors, and itchy eyes. They also underwent full ocular exploration, including IOP measurement, determination of best visual acuity with optical correction, gonioscopy and eye fundus examination.

### Human tissue samples

A human eye from a 45-year-old Caucasian female donor (cadaver) with no reported ocular pathology was obtained from the National Disease Research Interchange (Philadelphia, PA). The eye was fixed with 4% paraformaldehyde in 0.1 phosphate buffer (pH7.2) and embedded in paraffin as previously reported^37^. Histological cryostat sections (10 μm) were deparaffinized for immunohistochemical analysis.

### Animals

Wild-type Tü zebrafish (*Danio rerio*) were maintained at 28 °C with a 14 hours on/10 hours off light cycle and were fed a standard diet according to established protocols^38^. Zebrafish embryos were raised at 28 °C in E3 medium (5 mM NaCl; 0.17 mM KCl; 0.33 mM CaCl_2_; 0.33 mM MgSO_4_, and 0.0001% methylene blue, pH 7.2). A total of 100 μM phenylthiourea (PTU) was applied to embryos from 22 hpf to prevent melanization. All animal husbandry and experiments were approved and conducted in accordance with the guidelines set forth by the Institutional Animal Research Committee of the University of Castilla-La Mancha (approval number PR-2015-04-10). Zebrafish embryos and larvae were anaesthetized with 0.02% MS 222 and immobilized in a 2% methylcellulose solution for imaging.

### Exome capture, sequencing and analysis

Blood genomic DNA samples (3 μg) from 26 unrelated probands were processed for WES at the Centro Nacional de Análisis Genómico (CNAG-CRG, Barcelona, Spain). Joint exome capture was carried out for 20 samples using the SeqCap EZ Exome Enrichment Kit v3.0 (Roche NimbleGen) and for the remaining six samples (PCG-86, PCG-118, PCG-181, PCG-192, PCG-205 and PCG-208) using the SureSelect Human All Exon v4 kit (Agilent Technologies) employing a modified manufacturer’s protocol as described in the Supplementary Methods section. Paired-end sequencing was performed on a HiSeq2000 instrument (Illumina) as described in the Supplementary Methods section, using 2 × 76 bp reads (58X-116X mean coverage) or 2 × 101 bp reads (75-96X mean coverage) for each of the two kits employed, respectively. Exome data analysis was carried out as indicated in the Supplementary Methods section.

### Variant selection and validation

Initially, the common single-nucleotide polymorphisms (SNPs), defined as those with minor allele frequency ≥1% according to reported frequencies in the 1000 Genomes Project (phase 1, 08-04-2010 dataset) and in 5400 individuals from the National Heart, Lung, and Blood Institute (NHLBI) Exome Sequencing Project (all ethnicities) were filtered out. Selected variants included truncating mutations (nonsense and indels producing a frameshift), splice sites and missense variants. We selected only missense variants predicted to be pathogenic by SIFT (Sorting Intolerant From Tolerant)^39^ or PolyPhen (Polymorphism Phenotyping)^40^. The selected variants were validated by Sanger sequencing. The detailed variant filtering workflow followed in this study is summarized in Fig. 1A and B.

### Variant screening in congenital glaucoma patients by Sanger sequencing

The genomic DNA was extracted from peripheral blood, using the *QIAamp DNA Blood Mini Kit*. To screen *GPATCH3* variants by automatic Sanger sequencing in two cohorts of congenital glaucoma patients, the proximal promoter (nucleotides -1 to -326) and the seven exons of this gene were amplified by PCR using the primers described in Supplementary Table 2. The variants identified in each subject were confirmed by sequencing a new amplification product.

### Site-directed mutagenesis and cloning of the *GPATCH3* variants

Two *GPATCH3* variants (p.Asn234Ser and p.Gly475Glu), identified in this study, were obtained by site-directed mutagenesis using the QuickChange Lightning Site-Directed Mutagenesis Kit (Agilent Technologies, Santa Clara, CA, USA), as indicated in the Supplementary Methods section. The different recombinant GPATCH3 versions were transiently expressed in human embryonic kidney 293T (HEK-293T), which were cultured as indicated in the Supplementary Methods section. Transient plasmid transfections were carried out with 50–500 ng of plasmid DNA using the Superfect Transfection Reagent, according to the manufacturer’s instructions.

### Transactivation assays

GPATCH3 transactivation assays were performed using the Luciferase Assay System (Promega) according to the manufacturer’s instructions. HEK-293T cells were transfected with 500 ng of the recombinant GPATCH3 expression vector [pcDNA3.1(-) myc-his], along with 50 ng of the recombinant pGL3-basic-*CXCR4* luciferase reporter and 50 ng of the recombinant CYP1B1 expression vector [pcDNA3.1(-) myc-his] (transfection control) as indicated in the Supplementary Methods section. Recombinant expression vectors [pcDNA3.1(-) myc-his] encoding either the transcription factor FOXC2 or the secreted protein myocilin were used as positive and negative controls of the assay, respectively. Finally, the cell lysates were assayed for firefly luciferase activity.

### Western blotting and antibodies

For the Western blot analyses, the tissue extracts, prepared as indicated in the Supplementary Methods section, were fractionated by sodium dodecyl sulphate-polyacrylamide gel electrophoresis (SDS-PAGE) using the Mini-PROTEAN III Gel Electrophoresis System as previously described^41^. The endogenous GPATCH3 protein present in different tissue extracts was detected using a commercial rabbit polyclonal antibody (HPA032078, Sigma) as the primary antibody, diluted to 1:1000. Horseradish peroxidase-conjugated antibodies against rabbit IgG (#1858415, Pierce) were diluted to 1:1000–1:4000. We used a commercial mouse monoclonal anti-myc antibody (sc-40, Santa Cruz Biotechnology) to detect myc-tagged recombinant proteins, diluted at 1:250. Horse-radish peroxidase-conjugated antibodies against mouse IgG (#32430 ThermoScientific) were diluted to 1:500–1:1000. Chemiluminescence detection and the densitometry for protein band quantification were performed as previously described^41^. As an additional sample loading control, we detected the endogenous lactate dehydrogenase (LDH) protein in cell extracts using a goat anti-LDH antibody diluted to 1:5000 (AB1222, Chemicon) and an anti-goat IgG horseradish peroxidase-conjugated antibody (sc-2033, Santa Cruz Biotechnology), diluted to 1:2000.

### Fluorescent-whole mount *in situ* hybridization (FWISH)

The templates for *gpatch3* riboprobes were amplified from a commercial *gpatch3* cDNA (#8146986, Source Bioscience) and cloned into pCRII-TOPO plasmid (primer sequences: 5′-ATTGTGATTTATTCCCGTATAAGACGA-3′ and 5′-CCTGATTTGTTTGTGTTGTTGTG-3′). Sense and antisense riboprobes were transcribed from linearized plasmid using either T7 or Sp6 RNA polymerase and were fluorescently labeled using a FISH Tag RNA Green Kit. Embryos were dechorionated and fixed in 2% sweet paraformaldehyde [2% PFA, 4% sucrose, 10 mM phosphate-buffered saline (PBS) pH 7.3] overnight at 4 °C and then dehydrated and stored in 100% methanol. Proteinase-K permeabilized embryos were prehybridized in hybridization mix [50% v/v deionized formamide, 5X saline sodium citrate Buffer (SSC) (750 mM NaCl, 75 mM Na_3_Citrate), 5 mg/ml tRNA, 50 μg/ml heparin, and 0.1% Tween-20, pH 6.0] at 55 °C for 4 hours and then hybridized with 100 ng of fluorescent riboprobe overnight. SSC washed embryos were oriented and mounted in Fluorescent Mounting Medium and visualized in an LSM710 Zeiss confocal microscope. Fluorescence emitted by Alexa-488-conjugated riboprobes and embryo autofluorescence was registered at 495–529 nm and 553–677 nm, respectively. Z-Stack maximum intensity projections of embryos were obtained with ZEN software (Zeiss).

### Fluorescent-whole mount immunohistochemistry (FWIHC)

Embryos were dechorionated and fixed in 2% sweet paraformaldehyde (2% PFA, 4% sucrose, and 10 mM PBS pH 7.3) overnight at 4 °C and then dehydrated and stored in 100% methanol. Proteinase-K permeabilized embryos were blocked in IB [10% FBS, 1% dimethyl sulfoxide (DMSO), and 1% Triton-X100 in DPBS] for 1 hour at room temperature and incubated with rabbit anti-GPATCH3 1:100 primary antibody (HPA032078, Sigma) and Cy2 donkey anti-rabbit 1:1000 secondary antibody (Jackson ImmunoResearch) overnight at 4 °C. Washed embryos (1% Triton X-100 in PBS) were counterstained with DAPI, oriented and mounted in Fluorescent Mounting Medium and visualized using an LSM710 Zeiss confocal microscope. Fluorescence emitted by the Cy2-conjugated antibody and embryo autofluorescence was registered at 490–518 nm and 553–677 nm respectively. Z-Stack maximum intensity projections of embryos were obtained with ZEN software (Zeiss).

### Gelatin embedding and cryosectioning

Sectioned adult zebrafish heads or dechorionated embryos were fixed in 2% sweet paraformaldehyde (2% PFA, 4% sucrose, and 10 mM PBS pH 7.3) overnight at 4 °C and were cryoprotected by immersion in 30% sucrose in PBS for 3 days at 4 °C. Adult zebrafish heads and embryos were embedded and oriented in OCT medium or 10% porcine gelatin with 15% sucrose, respectively, and stored at −80 °C. Seriated sections (10 μm) were obtained in a Leica CM3050S cryostat and stored at −80 °C for further use.

### Immunohistochemistry and histology

Cryosections were blocked in IB (10% FBS, 1% DMSO, and 1% Triton X-100 in DPBS) for 1 hour at room temperature and incubated with rabbit anti-GPATCH3 1:100 primary antibody (HPA032078, Sigma) and Cy2 donkey anti-rabbit 1:1000 secondary antibody (Jackson ImmunoResearch) overnight at 4 °C. Then, cryosections were counterstained with DAPI, mounted in Fluorescent Mounting Medium and visualized using an LSM710 Zeiss confocal microscope. Fluorescence emitted by DAPI and the Cy2-conjugated antibody and embryo autofluorescence was registered at 411–464 nm, 490–518 nm and 553–677 nm respectively. Hematoxylin and eosin staining was performed according to standard protocols.

### Morpholino microinjection

*Gpatch3* knockdown was performed using either translation-blocking (*gpatch3*^*ATG*^) or splicing-blocking (*gpatch3*^*ex1/2*^or *gpatch3*^*ex2/3*^) antisense morpholino oligonucleotides (MO) purchased from Gene Tools, LLC. The MO sequences were *gpatch3*^*ATG*^: GCCGCCATACTGGAGTCTGTGTAAC, *gpatch3*^*ex1/2*^: TCTTTTATTTTGTCACTCACCGCTC and *gpatch3*^*ex2/3*^: TCATCCTGAAACACACAAACACACT. Embryos were injected in the yolk with 2.0 ng of MO at the 1–2 cell stage. For rescue experiments, 2.0 ng of the *gpatch3*^*ATG*^ MO were co-injected with 0.4 ng of *gpatch3* mRNA. Two nanograms of a MO that targets a human beta-globin intron mutation that causes beta-thalassemia (CCTCTTACCTCAGTTACAATTTAT), was used as a standard negative control and acquired from Gene Tools. For *gpatch3* overexpression, 0.4 ng of mRNA were also microinjected in the embryo’s yolk at the 1–2 cell stage.

### *In silico* analyses

*In silico* analyses of *GPATCH3* sequences and their different variants were carried out using the programs described in the Supplementary Methods section. Variants were named using directions from Mutalyzer (https://mutalyzer.nl/), according to RefSeq NM_022078.2. The *GPATCH3* transcription start site and 5′-UTR sequence were defined according to Ensembl ENST00000361720. The first nucleotide of the translation initiation site was numbered as nucleotide +1.

### Statistical analysis

The statistical comparisons between groups were performed using either the *t*-test or the one-way analysis of variance (ANOVA). A Bonferroni correction was applied to adjust tests for multiple comparisons. Statistical analysis of the data was performed using the SigmaStat 2.0 software (SPSS Science Inc., Inc., Chicago, IL, USA).

## Additional Information

**How to cite this article:** Ferre-Fernández, J.-J. *et al*. Whole-Exome Sequencing of Congenital Glaucoma Patients Reveals Hypermorphic Variants in *GPATCH3*, a New Gene Involved in Ocular and Craniofacial Development. *Sci. Rep.* **7**, 46175; doi: 10.1038/srep46175 (2017).

**Publisher's note:** Springer Nature remains neutral with regard to jurisdictional claims in published maps and institutional affiliations.

## Supplementary Material

Supplementary Information

## Figures and Tables

**Table 1:** *GPATCH3* variants identified in congenital glaucoma patients.

Patient	Chromosome position (GRCh38.p2)	Nucleotide change (rs# or ss#)	Aminoacid change	Frequency in ExAC (%)	SIFT^a^/PolyPhen^b^ score	HSF 3.0 predicted effects (# of algorithms)
WES group (n = 26)						
PCG-99	1:26897476 1:26891164	c.701A > G (rs35243557) c.1424G > A (rs376709877)	p.Asn234Serp.Gly475Glu	0.46 0.0016	0.15/0.7 0.29/0.97	—
PCG cohort (n = 130)						
PCG-30	1:26900595	c.−129C > A (ss2031476744)	—	N.I.	—	—
	1:26891208	c.1380A > G (rs35647061)	p.Leu460=	0.13	—	ESEB (4)
PCG-116	1:26897476	c.701A > G (rs35243557)	p.Asn234Ser	0.46	0.15/0.7	—
PCG-145	1:2690037	c.67G > A (rs764461662)	p.Val23Met	0.002	—	—
PCG-180 PCG-205	1:26893277	c.1111 + 112G > A (rs116417426)	—	0.0044^c^	—	ESSB (2), ESEC (3) & CSSA (2)
PCG-F203	1:26900660	c.−194 C > G (rs554585879)	—	N.I.	—	—
V-0638	1:26892473	c.1299C > T (rs767504926)	p.Cys433 =	0.001		ESEB (4)
Secondary congenital glaucoma cohort (n=40)						
MOC-F5	1:26900760	c.−298_−295delGAGG (ss2031476745)	—	N.I.	—	—
	1:26890747	c.*263C > T (rs147217101)	—	1.3^c^	—	—
	1:26890736	c.*274C > T (rs115936179)	—	0.3^c^	—	—
MOC-F7	1:26893315	c.1111 + 74A > T (ss2031476742)	—	N.I.	—	—
ANF-0064-1 ANF-0064-2 (twins)	1:26900034	c.409C > T (ss2031476743)	p.Arg137Cys	N.I.	0.04/0.357	—

^a^Ranges from 0 to 1, predicted damaging if the score is ≤0.05 and tolerated if the score is >0.05. ^b^Ranges from 0 to 1. Score values are interpreted as follows: 0.0–0.15, benign; 0.15–0.85, possibly damaging; 0.85–1.0, damaging. ^c^1000 Genomes Project (http://www.internationalgenome.org/data). N.I.: Not identified. ESEB: Exonic Splicing Enhancer Broken. ESSB: Exonic Splicing Silencer Broken. ESEC: Exonic Splicing Enhancer Created. CSSA: Cryptic Splicing Site Sctivated. ExAC: Exome Aggregation Consortium (http://exac.broadinstitute.org/). HSF: Human Splicing Finder software (http://www.umd.be/HSF3/HSF.html). PolyPhen: Polymorphism Phenotyping (http://genetics.bwh.harvard.edu/pph/data/). SIFT: Sorting Intolerant From Tolerant (http://sift.bii.a-star.edu.sg/).
