# Supplementary material for: Whole-Exome Sequencing of Congenital Glaucoma Patients Reveals Hypermorphic Variants in GPATCH3, a New Gene Involved in Ocular and Craniofacial Development
Source: Sci Rep. 2017 Apr 11;7:46175. doi: 10.1038/srep46175 (PMC5387416; doi:10.1038/srep46175)
Supplement: Supplementary Information [file srep46175-s1.pdf]

**Supplementary Information for:**

# Whole-Exome Sequencing of Congenital Glaucoma Patients Reveals Hypermorphic Variants in *GPATCH3*, a New Gene Involved in Ocular and Craniofacial Development

Jesús-José Ferre-Fernández[1], José-Daniel Aroca-Aguilar, PhD,[1,2] Cristina Medina-Trillo, PhD,[1,2] Juan-Manuel Bonet-Fernández, PhD,[1,2] Carmen-Dora Méndez-Hernández, MD, PhD,[2,3] Laura Morales-Fernández, MD, PhD,[2,3] Marta Corton, PhD[4] María-José Cabañero-Valera[1,2], Marta Gut, PhD[5,6], Raul Tonda, PhD[5,6], Carmen Ayuso, MD, PhD,[4] Miguel Coca-Prados, PhD[7,8], Julián García-Feijoo, MD, PhD,[2,3] and Julio Escribano, PhD[1,2,*]

[1]Área de Genética, Facultad de Medicina/Instituto de Investigación en Discapacidades Neurológicas (IDINE), Universidad de Castilla-La Mancha, Albacete, Spain
[2]Cooperative Research Network on Age-Related Ocular Pathology, Visual and Life Quality, Instituto de Salud Carlos III, Madrid, Spain
[3]Servicio de Oftalmología, Hospital San Carlos, Madrid, Spain/Instituto de Investigación Sanitaria del Hospital Clínico San Carlos, Madrid, Spain
[4]Área de Genética & Genómica, Instituto de Investigación Sanitaria-Hospital Universitario Fundación Jiménez Díaz-Universidad Autónoma de Madrid (IIS-FJD, UAM), Madrid, Spain/Centro de Investigación Biomédica en Red de Enfermedades Raras (CIBERER), Madrid, Spain
[5]CNAG-CRG, Centre for Genomic Regulation (CRG), Institute of Science and Technology (BIST), Centre for Genomic Analysis (CNAG), Barcelona, Spain
[6]Universitat Pompeu Fabra (UPF), Barcelona, Spain
[7]Fundación de Investigación Oftalmológica, Instituto Oftalmológico Fernandez-Vega, Oviedo, Spain
[8]Department of Ophthalmology and Visual Science, Yale University School of Medicine, New Haven, CT, USA

[*]**Corresponding autor:** Julio Escribano, PhD, Área de Genética, Facultad de Medicina, Avda. de Almansa, 14, 02006-Albacete, Spain. E-Mail: julio.escribano@uclm.es

## Supplementary Methods

**Exome capture and sequencing.** For whole exome capture the SeqCap EZ Exome Enrichment Kit v3.0 (Roche NimbleGen), based on the human genome build GRCh37/hg19 with a 64.19 Mb sequence capture design, was used following a modified manufacturer's protocol with pre-capture multiplexing adapted to the Sciclone Liquid Handling System. In short, after Covaris E210 fragmentation of genomic DNA (1 μg) the Illumina TruSeq DNA libraries were prepared on the robotic system using Gold Standard DNASeq protocol. Adapter ligated DNA fragments were enriched by eight cycles of pre-capture PCR. Samples were manually pre-capture pooled with a combined mass of 750 ng for the baits hybridization step (47°C for 71 hours). After washes, the captured multiplexed library was amplified with 18 cycles of post-capture PCR. Size, concentration and quality of the captured material were determined using an Agilent DNA 7500 chip. The success of the enrichment was measured by qPCR SYBR Green assay on a Roche LightCycler 480 Instrument evaluating one specific genomic *locus* within the amplified sample library and amplified captured DNA. The three sample pools were sequenced on an Illumina HiSeq 2000 instrument in one sequencing lane following the manufacturer's protocol, with a paired end run of 2x76 bp to reach a mean coverage of 59X-116X of the approximately 64.2 Mb target region.

The library preparation for capturing of selected DNA regions with SureSelect XT Human All Exon V4 kit (Agilent Technologies) based on the CCDS, RefSeq and UCSC version from March 2011, with 51.19 Mb targeted capture of coding exons, was performed according to Agilent's SureSelect protocol for Illumina paired-end sequencing. In brief, genomic DNA (3.0 μg) was sheared on a Covaris E210 instrument followed by Agilent adaptor ligation. The adaptor-modified DNA fragments (150-300 bp) were PCR amplified (six cycles using Herculase II fusion DNA polymerase) with SureSelect Primer and SureSelect Pre-Capture Reverse PCR primers (Agilent technologies) and hybridized to SureSelect baits (Agilent Technologies) for 24 hours at 65°C, using an Applied Biosystems 2720 Thermal Cycler. After the washes the eluate was PCR amplified (16 cycles) in order to add the index tags using Sure SelectXT Indexes for Illumina (Agilent Technologies). The final library size and concentration and sequencing were carried out as described previously with a paired end run of 2x101 bp to reach mean coverage of 7596X of the approximately 51.2 Mb target region. Image analysis, base calling and quality scoring of all the sequencing runs were processed using the manufacturer's software Real Time Analysis (RTA 1.13.48) and followed by generation of FASTQ sequence files by CASAVA.

**Exome data analysis.** Sequencing reads were trimmed from the 3' end up to the first base with a Phred quality >9 and were mapped to the Human reference genome (GRCh37) using GEM[1] to generate BAM files containing only properly paired and uniquely mapped reads (see coverage on the target regions per sample in Supplementary Table 1; available at www.aaojournal.org). The files were further processed with Picard tools (http://picard.sourceforge.net/) to remove duplicates, and local realignment was performed with the Genome Analysis Tool Kit (GATK). SAMtools[2] was used on the processed BAM files to call single nucleotide variants (SNVs) and small insertion deletions (InDels). Functional annotations from Ensembl (http://www.ensembl.org/index.html) were added to the resulting VCF using snpEff[3]. snpSift[4] was used to add information from dbSNP[5], the 1000 Genomes Project[6], the NHLBI Exome Sequencing Project and a variety of conservation and deleteriousness predictions included in dbNSFP[7].

**Site-directed mutagenesis and cloning of *GPATCH3* mutations.** The wild-type *GPATCH3* coding sequence obtained from a commercial cDNA (Origene, NM_022078, SC122923) was subcloned into the XbaI and BamHI restriction sites of the modified mammalian cell expression vector pcDNA3.1(-) (Cterminal Myc-Tagged)[8] by directional PCR using the following primers:
5'AATT***TCTAGA***ATGGCGGTGCCCGGCGAG3' (P1) and
5'AA***GGATCC***AGGTCAGGCAATGAGGGGCT3' (P2) (XbaI and BamHI sequences are indicated in bold, respectively). This cDNA construct was used as a template to obtain the *GPATCH3* variants (p.Asn234Ser and p.Gly475Glu) by site-directed mutagenesis using the QuickChange Lightning SiteDirected Mutagenesis Kit (Agilent Technologies), with the primers and PCR conditions indicated in the Supplementary Table 3**,** according to the manufacturer's instructions. All site-directed mutagenesis products were sequenced to confirm the presence of the mutation and were subcloned into either the XbaI and BamHI restriction sites of pcDNA3.1(-).

**Transactivation activity assays.** A 600-bp fragment of the human CXCR4 gene promoter, which contains one Fox Binding Element, was cloned into the NheI/NcoI restriction sites of the pGL3-basic vector (Promega) by directional PCR using normal human genomic DNA as previously described.[9] HEK293T cells in 24-well tissue culture plates ($2x10^5$ cells/well) were transfected with 500 ng of either the different GPATCH3 variants expression vectors, the FOXC2 expression vector [pcDNA3.1(-) myc-his] as a positive control or the MYOC

expression vector [pcDNA3.1(-) myc-his] as a negative control, along with 25 ng of the recombinant pGL3-basic-CXCR4 luciferase reporter and 50 ng of the CYP1B1 expression vector [pcDNA3.1(-) myc-his] as a transfection control. The transactivation assays were performed 24 hours after the transfection. The cells were harvested and assayed for firefly luciferase activity using the Luciferase Assay System according to the manufacturer's instructions. GPATCH3, FOXC2 (positive control), myocilin-HA (MYOC, negative control) and CYP1B1-myc (transfection control) recombinant proteins and endogenous lactate dehydrogenase (LDH, loading control) were detected by Western blot.

**Culture of HEK-293T cells.** The cells were maintained in Dulbecco's modified Eagle's medium (DMEM) supplemented with 10% fetal bovine serum (FBS), 1% L-glutamine and antibiotics (Normocin, Invitrogen) at 37°C in a fully humidified 5% $CO_2$ atmosphere.

**Immunocytochemistry.** The HEK-293T cells were seeded on coverslips placed into 24-well plates were transiently transfected with DNA constructs encoding FOXC2 and different GPATCH3 variants. All of the transfections were performed using the SuperFect Transfection Reagent. After the transfection, the cells were washed once with DPBS (Dulbecco's Phosphate-Buffered Saline) and cultured for 24 hours. The cells were then fixed with 4% paraformaldehyde for 10 min at room temperature, followed by incubation with phosphate buffered solution containing 0.2% Triton X-100, 10% fetal bovine serum (FBS), and 5% bovine serum albumin for 30 min at room temperature. The recombinant proteins were detected using an anti-myc antibody (Santa Cruz Biotechnology) (at a 1:500 dilution) at 4°C overnight followed by a Cy2-conjugated anti-mouse IgG (Jackson ImmunoResearch Labs,1:1000 dilution) for 2 hours at room temperature. Finally, the coverslips were mounted on glass slides using polyvinyl alcohol mounting medium with DABCO containing DAPI (40,60-diamidino-2-phenylindole), and the cells were viewed under an LSM 710 Zeiss confocal microscope

**Bioinformatic analyses.** The deleterious effect of mutations was predicted on line with the Sorting Intolerant From Tolerant (SIFT),[10] and PolyPhen programs. The UniProt database was searched to identify protein domains. The Consite software was used to identify putative transcription factor target sequences[11]. The MicroSNiPer software was used to identify putative miRNA target sequences[12]. The effect of variants on splicing was predicted using the Human Splicing Finder software[13]. Amino acid sequence alignments were carried out with Clustalw[14].

**Preparation of tissue extracts for Western blot.** Human ocular tissues were obtained from enucleated eyes of dead donors through the National Disease Research Interchange (Philadelphia, PA, USA). Dissection was carried out, and tissues were collected in Eppendorf tubes. Proteins were extracted by mechanical pulverization of tissues in liquid $N_2$ as previously described[8]. Non ocular tissue extracts were obtained from BioChain Institute, Inc. and Santa Cruz Biotechnology, Inc. Zebrafish embryos (96 hpf) were dechorionated by pronase treatment (1 mg/ml) for 5 min at room temperature[15], and yolk sacks were manually removed under a light stereomicroscope SMZ18 (Nikon) using microdissection needles. Pools of 50 zebrafish embryos were homogenized in extraction buffer (Tris HCl pH 7.4 50 mM, IGEPAL 1%, NaCl 150 mM, EDTA 1 mM, PMFS 1 mM, Leupeptin 1 µg/µl, and NaF 1 mM). The extract was centrifuged, and the supernatant was frozen at -80°C. The protein content of each sample was quantitated using the Bicinchoninic Acid Protein Assay Kit (Thermo Scientific), following the manufacturer's recommendations.

**Alcian blue cartilage staining.** 96 hours post fecundation (hpf) MO injected and wild type larvae were fixed in 4% paraformaldehyde overnight. Larvae were washed with PBS-Tween (PBST) to remove paraformaldehyde, bleached in 3% hydrogen peroxide and 1% KOH solution to remove pigment, and then were washed again three times in PBST. Larvae were incubated overnight in an Alcian blue solution (0.1% Alcian blue, 5% HCl and 70% etanol), rehydrated in a staged ethanol series and treated with 50 mg/ml trypsin for 2 hours at 37ºC to dissolve brain tissue and allow visualization of pharyngeal arch cartilages. Bright-field images were taken using a Nikon SMZ18 microscope equipped with a Nikon DSRi2 digital camera.

**Confocal imaging of Tg(*sox10*:eGFP) reporter fishes.** *Gpatch3* knockdown of embryos obtained from Tg(*sox10*:eGFP) reporter fishes (kindly provided by Dra. Cavodeassi) was performed using the *gpatch3$^{ATG}$* morpholino oligonucleotide (MO) purchased from Gene Tools, LLC. Embryos were injected with 2.0 ng of MO at the 1-2 cell stage. Embryos (96 hpf) were anesthetized with 0,02% Tricaine (MS222) (Sigma), mounted in 2% methylcellulose (Sigma) and visualized using an LSM710 Zeiss confocal microscope. Fluorescence emitted by the GFP reporter protein was registered at 494-552 nm. Z-Stack maximum intensity projections of embryos were obtained with ZEN software (Zeiss).

**Supplementary References**


1 Marco-Sola, S., Sammeth, M., Guigó, R. & Ribeca, P. The GEM mapper: fast, accurate and versatile alignment by filtration. *Nat Methods* **9**, 1185-1188, doi:10.1038/nmeth.2221 (2012).

2 Li, H. *et al.* The Sequence Alignment/Map format and SAMtools. *Bioinformatics* **25**, 2078-2079, doi:10.1093/bioinformatics/btp352 (2009).

3 Cingolani, P. *et al.* A program for annotating and predicting the effects of single nucleotide polymorphisms, SnpEff: SNPs in the genome of Drosophila melanogaster strain w1118; iso-2; iso-3. *Fly (Austin)* **6**, 80-92, doi:10.4161/fly.19695 (2012).

4 Cingolani, P. *et al.* Using Drosophila melanogaster as a Model for Genotoxic Chemical Mutational Studies with a New Program, SnpSift. *Front Genet* **3**, 35, doi:10.3389/fgene.2012.00035 (2012).

5 Sherry, S. T. *et al.* dbSNP: the NCBI database of genetic variation. *Nucleic Acids Res* **29**, 308311 (2001).

6 Abecasis, G. R. *et al.* A map of human genome variation from population-scale sequencing. *Nature* **467**, 1061-1073, doi:10.1038/nature09534 (2010).

7 Liu, X., Jian, X. & Boerwinkle, E. dbNSFP v2.0: a database of human non-synonymous SNVs and their functional predictions and annotations. *Hum Mutat* **34**, E2393-2402, doi:10.1002/humu.22376 (2013).

8 Aroca-Aguilar, J., Sanchez-Sanchez, F., Ghosh, S., Coca-Prados, M. & Escribano, J. Myocilin mutations causing glaucoma inhibit the intracellular endoproteolytic cleavage of myocilin between amino acids Arg(226) and Ile(227). *Journal of Biological Chemistry* **280**, 21043-21051, doi:10.1074/jbc.M501340200 (2005).

9 Medina-Trillo, C. *et al.* Hypo- and hypermorphic FOXC1 mutations in dominant glaucoma: transactivation and phenotypic variability. *PLoS One* **10**, e0119272, doi:10.1371/journal.pone.0119272 (2015).

10 Kumar, P., Henikoff, S. & Ng, P. C. Predicting the effects of coding non-synonymous variants on protein function using the SIFT algorithm. *Nat.Protoc.* **4**, 1073-1081 (2009).

11 Sandelin, A., Wasserman, W. W. & Lenhard, B. ConSite: web-based prediction of regulatory elements using cross-species comparison. *Nucleic Acids Res* **32**, W249-252, doi:10.1093/nar/gkh372 (2004).

12 Barenboim, M., Zoltick, B. J., Guo, Y. & Weinberger, D. R. MicroSNiPer: a web tool for prediction of SNP effects on putative microRNA targets. *Hum.Mutat.* **31**, 1223-1232 (2010).

13 Desmet, F. O. *et al.* Human Splicing Finder: an online bioinformatics tool to predict splicing signals. *Nucleic Acids Res* **37**, e67, doi:10.1093/nar/gkp215 (2009).

14 Thompson, J. D., Higgins, D. G. & Gibson, T. J. CLUSTAL W: improving the sensitivity of progressive multiple sequence alignment through sequence weighting, position-specific gap penalties and weight matrix choice. *Nucleic Acids Res.* **22**, 4673-4680 (1994).

15 Westerfield, M. *The Zebrafish Book: A Guide for the Laboratory Use of Zebrafish (Danio rerio)*. 5th edn, (Eugene: University of Oregon Press, 2000).

**Supplementary Table1. Coverage ≥10 of each WES sample**.

| Proband | c10[a] | Proband | c10[a] |
|---|---|---|---|
| **PCG-41** | 71.98 | **PCG-118** | 93.31 |
| **PCG-60** | 72.41 | **PCG-134** | 81.18 |
| **PCG-65** | 79.20 | **PCG-148** | 73.13 |
| **PCG-66** | 78.81 | **PCG-150** | 77.22 |
| **PCG-78** | 75.62 | **PCG-161** | 80.71 |
| **PCG-86** | 95.01 | **PCG-163** | 84.51 |
| **PCG-94** | 78.55 | **PCG-165** | 83.07 |
| **PCG-96** | 73.29 | **PCG-170** | 86.23 |
| **PCG-97** | 74.84 | **PCG-181** | 94.36 |
| **PCG-99** | 80.86 | **PCG-183** | 82.54 |
| **PCG-101** | 80.63 | **PCG-192** | 94.14 |
| **PCG-104** | 82.51 | **PCG-205** | 94.37 |
| **PCG-113** | 83.64 | **PCG-208** | 95.38 |

[a]c10: percentage of the mappable exome covered by at least 10 uniquely mapped and properly paired (according to orientation and distance) read pairs. Note that six samples (PCG-86, PCG-118, PCG-181, PCG-192, PCG-205 and PCG-208) were captured with Agilent SureSelect XT Human All Exon v4 (54Mb) and the rest with Nimblegen SeqCap EZ Exome Enrichment Kit v3.0 (64Mb).

**Supplementary Table 2. Primer sequences and thermal cycling conditions used for *GPATCH3* amplification**

| Primer set | Sequence (5'→3') | Annealing temperature (°C)/time (s) | PCR cycles | Amplicon length (bp) |
|---|---|---|---|---|
| **PROM-E1** | F: ATGGAAGAACGACTAAAGGTTCAGA R: GAGTCCTAACCAGAGGCCTGACT | 56/90 | 40 | 900 |
| **E2** | F: CACTGTGTCGGCCTAGATTTTTACT R: AGCTCTGGACCATCTCCCTACA | 56/80 | 40 | 764 |
| **E3** | F: GGGCCAAATATGTGCTGCTT R: ACAGGGAACCCCAAAAGAATG | 56/30 | 40 | 380 |
| **E4** | F: ATGGTGGCATGCACCTGTAG R: AAGGATGAAGGCTCTGTCCAAA | 58/60 | 42 | 483 |
| **E5-6** | F: GAAACCTGATTCTTCCCCCTTCT R: AGCGCTAGGATTACAGGCTTGA | 56/60 | 40 | 625 |
| **E7-3'UTR** | F: GCTGGAGAACAAAGACCCAGAT R: TCGCCCAGGTCCCTTTC | 56/60 | 40 | 730 |

F: forward; R: reverse.

**Supplementary Table 3. Primer sequences and PCR conditions used for *GPATCH3* site-directed mutagenesis.**

| Primer set | Sequence (5'→3') | Annealing temperature (°C)/time (s) | PCR cycles |
|---|---|---|---|
| **p.Asn234Ser** | F: CCCGGCGCTACGGCAGTGTGCCTTTTGAG R: CTCAAAAGGCACACTGCCGTAGCGCCGGG | 58/15 | 30 |
| **p.Gly475Glu** | F: CGTAGAAATGGCTTGGAGCTCATCTCCACCATC R GATGGTGGAGATGAGCTCCAAGCCATTTCTACG | 58/15 | 30 |

F: forward; R: reverse.

**Supplementary Table 4. Clinical features of congenital glaucoma patients with *GPATCH3* rare variants.**

| Patient | Phenotype | Sex/laterality | Age at diagnosis | IOP (mmHg) at diagnosis (OD/OI) | C/D (OD/OS) | Number and type of surgical interventions (OD/OS) |
|---|---|---|---|---|---|---|
| PCG-99 | PCG, C | Male/U | 3 months | NA | NA | G/G |
| PCG-30 | PCG | Male/B | Birth | 23/28 | | G/G |
| PCG-116 | PCG | Female/U(OD) | 3 months | 32/- | 0.7/- | 2G/- |
| PCG-145 | PCG | Female/B | 6 years | ND | 1.0/1.0 | -/T |
| PCG-180 | PCG | Male/B | 5 months | 28/30 | 0.3/EN | G/2G+3T+V+EN |
| PCG-203 | PCG | Male/B | 2 months | NA | NA | NA |
| PCG-205 | PCG | Male/B | 3 months | 30/35 | 1.0/1.0 | G+T+V/G+T+V |
| V-0638 | PCG | Male/B | 3 months | NA | NA | G/2G |
| MOC-F5 | CG+ICE | Male/U(OD) | 2 months | NA | 0.1/0.1 | none |
| MOC-F7 | CG+AN | Male/B | 4 months | NA | | 2T/T |
| ANF-0064-1/ | CG+ASD+ M | Female/B | Birth | 35/35 | NA | None |
| ANF-0064-2 (twins) | CG+ASD+M | Female/B | Birth | 35/35 | NA | None |

AN: aniridia; ASD: anterior segment dysgenesis; B/U: bilateral/unilateral; C: corectopia; C/D: cup/disk ratio; CG: congenital glaucoma; EN: enucleated; ICE: iridocorneal endothelial syndrome; M: microphthalmia; NA: Not available; OD/OS: right eye/left eye; G: goniotomy; T: trabeculectomy; V: valve.

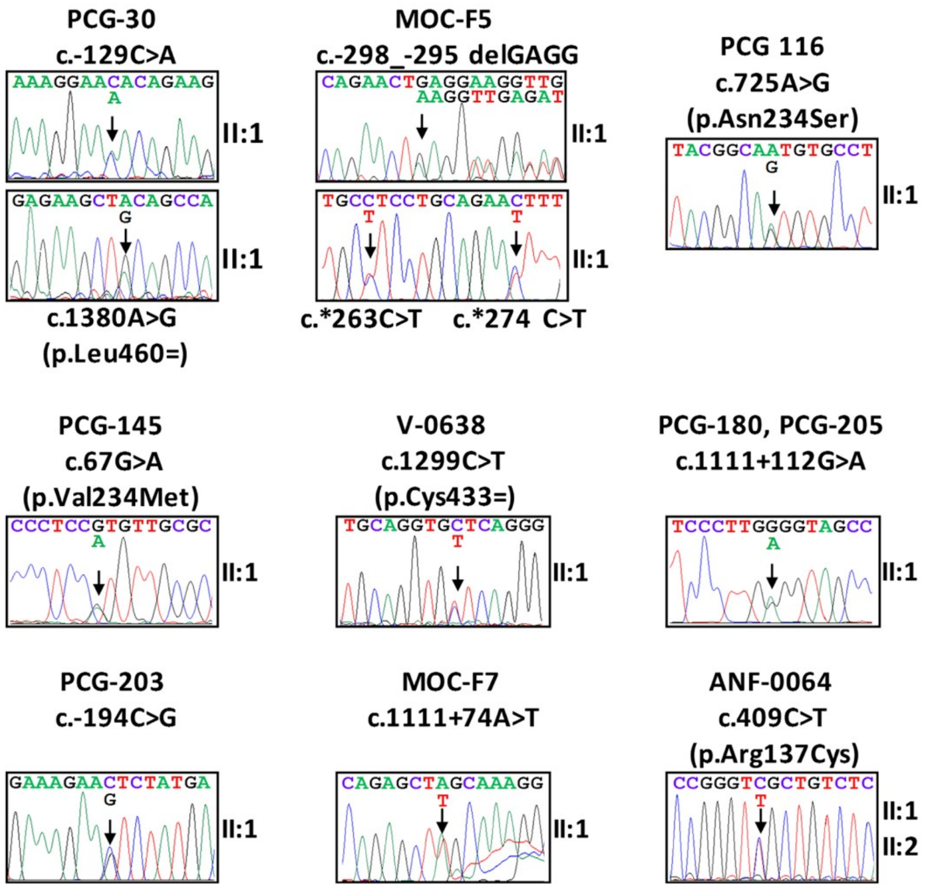


**Supplementary Figure 1. Electropherograms of the *GPATCH3* mutations identified in the study**.

Arrows in the electropherograms indicate the locations of mutations.

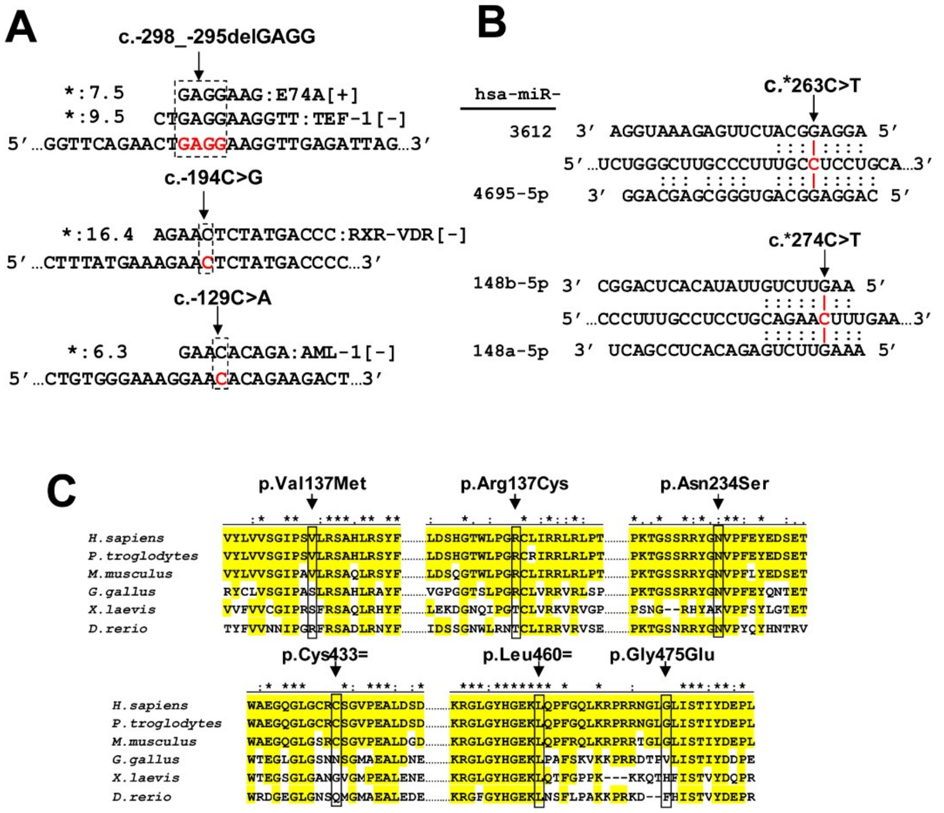


**Supplementary Figure 2. Bioinformatics analyses of the *GPATCH3* variants identified in congenital glaucoma patients.** (A) The promoter variants disrupt putative target sequences for different transcription factors predicted with the Consite software. Asterisks indicate the Consite Score of the predicted target. (B) The 3’-UTR variants disrupt putative target sequences for different miRNAs. The target miRNAs were predicted with the MicroSNiPer software. Dotted lines in the predicted miRNA target sequence indicate perfect Watson-Crick base pairing. The continuous vertical red line indicates the disruption of base pairing between the miRNA and the mutant target sequence. The mutant nucleotides are in red in panels (A) and (B). (C) Multiple alignments of amino acid sequences affected by mutations. The alignments were carried out with ClustalW[14]. The yellow background indicates the positions where the amino acids are identical.

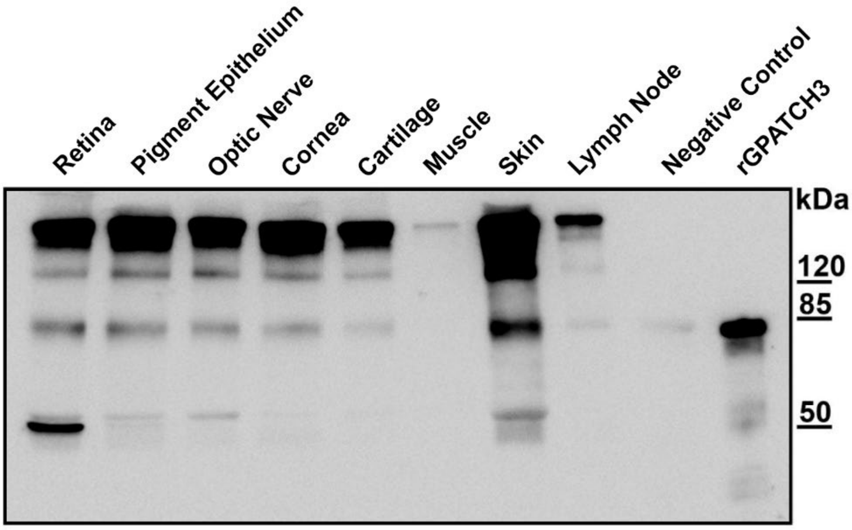


**Supplementary Figure 3. Western immunoblot analysis of the GPATCH3 protein present in human tissues.** Samples of human tissues: retina, pigment epithelium, optic nerve, cartilage and skin (20 µg of total protein) were prepared as described in the Materials and Methods section and analyzed by Western immunoblot. The GPATCH3 recombinant protein was transfected into HEK-293T cells and used as a positive control (rGPATCH3). Detection was carried out with the anti-GPATCH3 polyclonal antibody. The negative control consisted of a cell lysate from non-transfected HEK-293T cells.

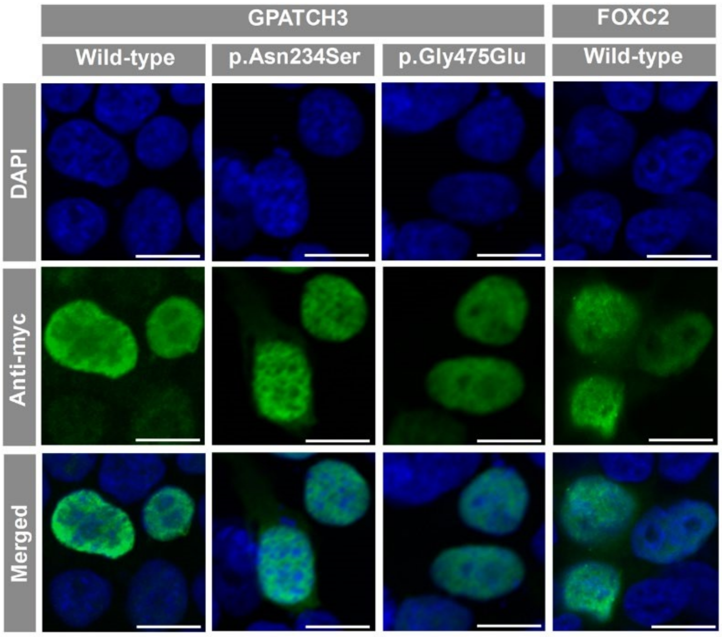


**Supplementary Figure 4. Nuclear localization of recombinant *GPATCH3* wild type and missense variants identified in PCG-F99.** cDNA constructs encoding the indicated recombinant versions of GPATCH3 and FOXC2 (positive control) were transiently expressed in HEK-293T cells. The recombinant proteins were detected via fluorescent immunocytochemistry using an anti-myc antibody (Santa Cruz Biotechnology). Nuclei were visualized using fluorescent DAPI staining. Horizontal bars correspond to 10 µm.

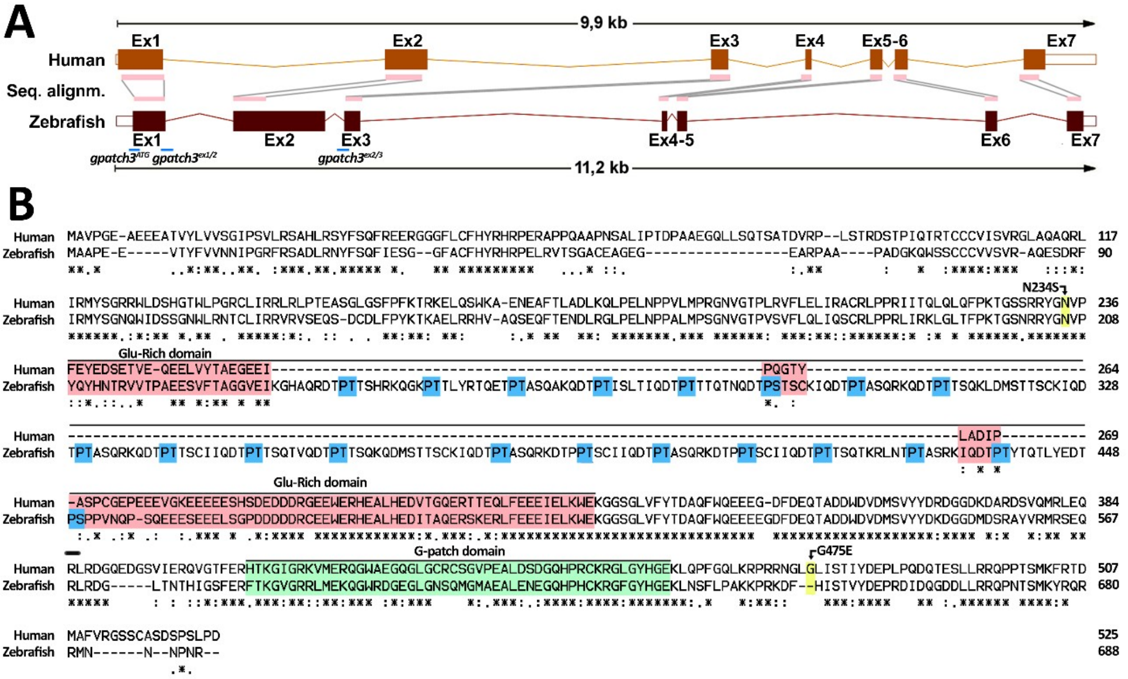


**Supplementary Figure 5. The *gpatch3* gene structure and amino acid sequence are highly similar to those of its human orthologue.** (A) Genomic alignment of human *GPATCH3* and zebrafish *gpatch3* genes. Note a non-conserved region in zebrafish exon 2. Pink rectangles indicate exonic conservation. Blue rectangles indicate the localization of the three morpholinos used in this study. (B) Amino acid sequence alignment of human and zebrafish GPATCH3 proteins. The locations of missense mutations present in patient PCG-99 are indicated and shaded in yellow boxes. The alignment was carried out with ClustalW[14]. The predicted Glu-rich and G-patch domains are indicated by pink and green backgrounds, respectively. The localization of 20 Pro-Thr/Ser repeats encoded by the zebrafish specific exon 2 sequence are labeled in blue. The asterisks indicate the positions where all the amino acids are identical, two vertical dots show amino acids with similar chemical properties and one dot denotes amino acid positions with weak chemical similarity.

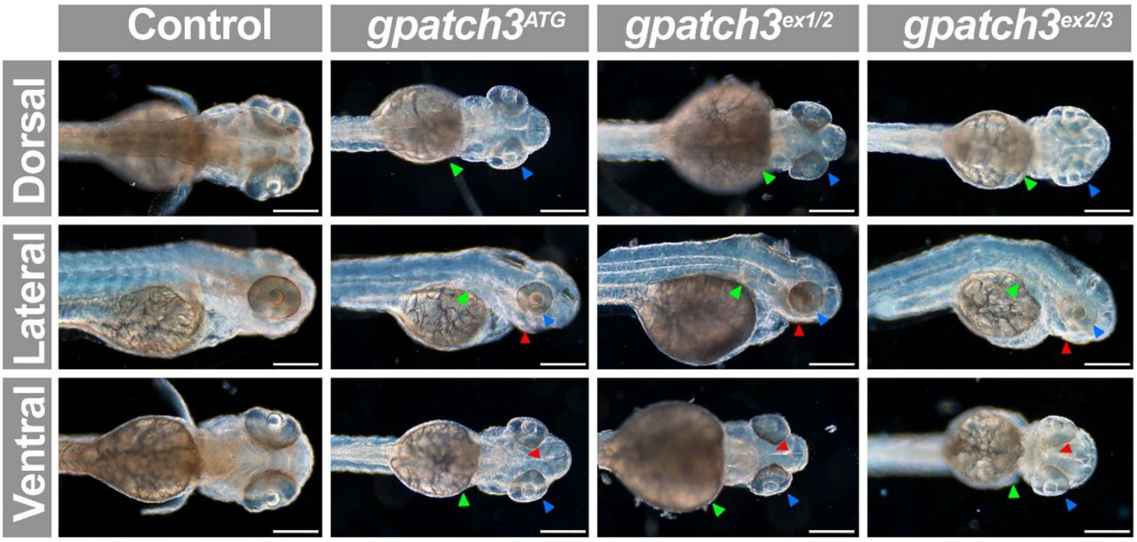


**Supplementary Figure 6. Zebrafish *gpatch3* morphants present similar gross morphological alterations with three different morpholinos.** Control, *Gpatch3*$^{ATG}$, *Gpatch3*$^{ex1/2}$ *and Gpatch3*$^{ex2/3}$ MOs (2.0 ng) were microinjected in 1-2 cells zebrafish embryos. The standard negative control consisted of a MO that targets a human beta-globin intron mutation that causes beta-thalassemia. At 72 hours post fecundation (hpf), larvae were analyzed. Dorsal, lateral and ventral dark field micrographs show altered phenotypes including microphthalmia (blue arrowheads) and abnormal development of pectoral fins (green arrowheads) and craniofacial structures including absence of jaw cartilages (red arrowheads). Scale bars indicate 200 μm.

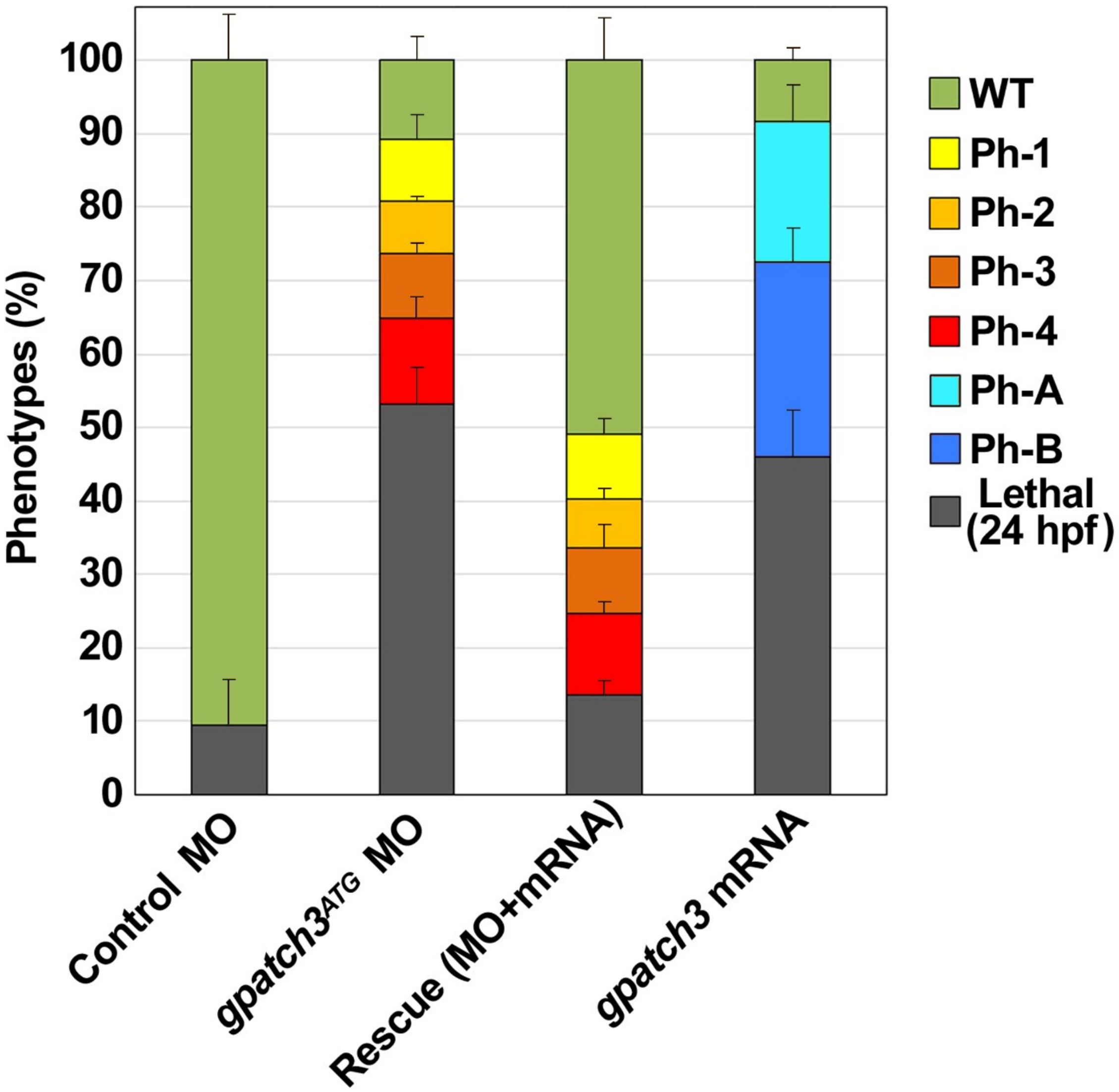


**Supplementary Figure 7. Proportion of zebrafish phenotypes observed in *gpatch3* knock-down, mRNA rescue and overexpression assays.** For gpatch3 *knockdown* zebrafish embryos were microinjected with 2.0 ng of either a control MO that targets a human beta-globin intron mutation that causes betathalassemia or the *gpatch3$^{ATG}$* MO. At 96 hpf, the proportion of phenotypes (Ph-1 to Ph-4, shown in Figs. 7 and 8) was calculated. mRNA rescue was carried out in zebrafish embryos co-injected with 2.0 ng of the *gpatch3$^{ATG}$* MO and 0.4 ng of *gpatch3* mRNA. At 96 hpf it was observed reduced lethality and increased wild-type-like phenotypes. For *gpatch3* overexpression assay embryos were microinjected in the yolk sack with 0.4 ng of *gpatch3* mRNA and at 96 hpf the proportion of phenotypes (Ph-A and Ph-B, shown in Figs. 9 and 10) was calculated. Values correspond to mean $\pm$ SEM of triplicate experiments with 60 to 200 embryos per experiment.

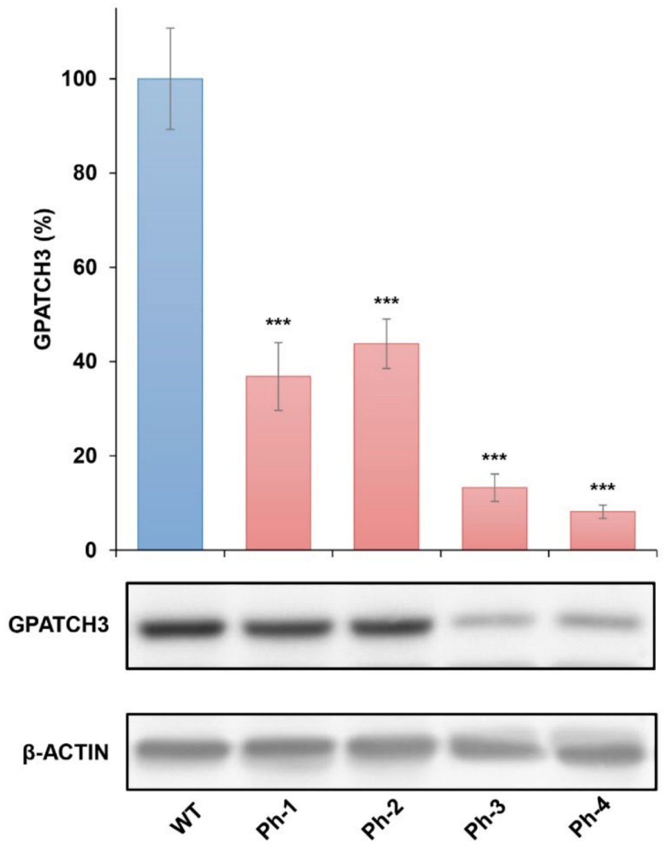


**Supplementary Figure 8. Morpholino microinjection silences the gpatch3 protein.** Quantitation by densitometry of the Gpatch3 protein detected by Western blot in 96 hpf zebrafish embryos microinjected with the *Gpatch3*$^{ATG}$ MO. 20 µg of total protein per sample were loaded for the analysis. The values were normalized with respect to β-actin in total lysates from the different morpholino phenotypes and are expressed as a percentage of the gpatch3 protein detected in wild-type animals. Values are expressed as mean ± SEM of quadruplicate experiments, *** $P < 0.001$ (ANOVA).

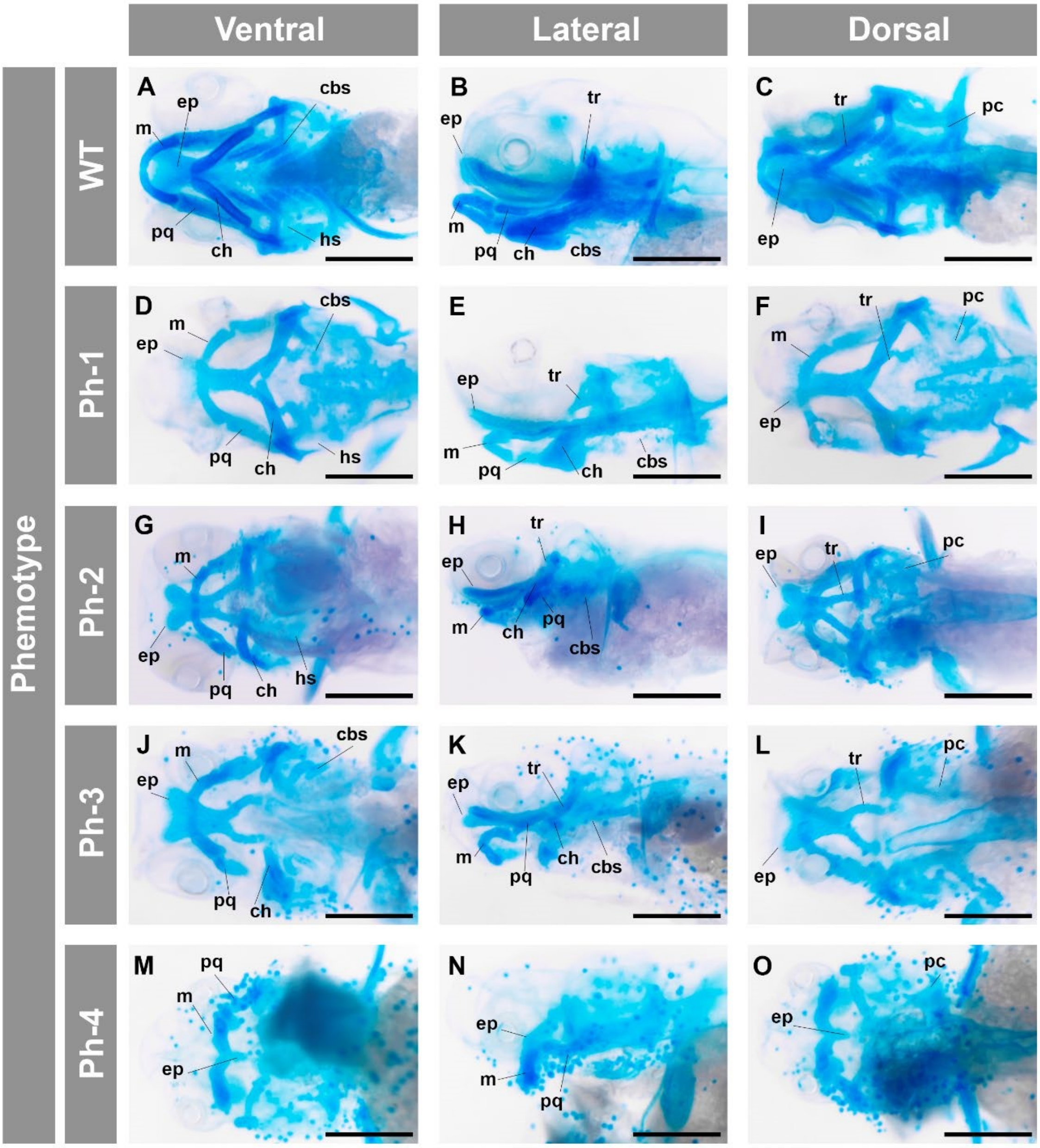


**Supplementary Figure 9. Alcian blue cartilage staining in *Gpatch3*$^{ATG}$ morphants at 96 hpf.** cbs, ceratobranchials; ch, ceratohyal, m, Meckel’s cartilage; ep, ethmoid plate; hs, hyosymplectic; pc, parachordal; pq, palatoquadrate; tr, trabecula. Scale bars represent 200 µm.

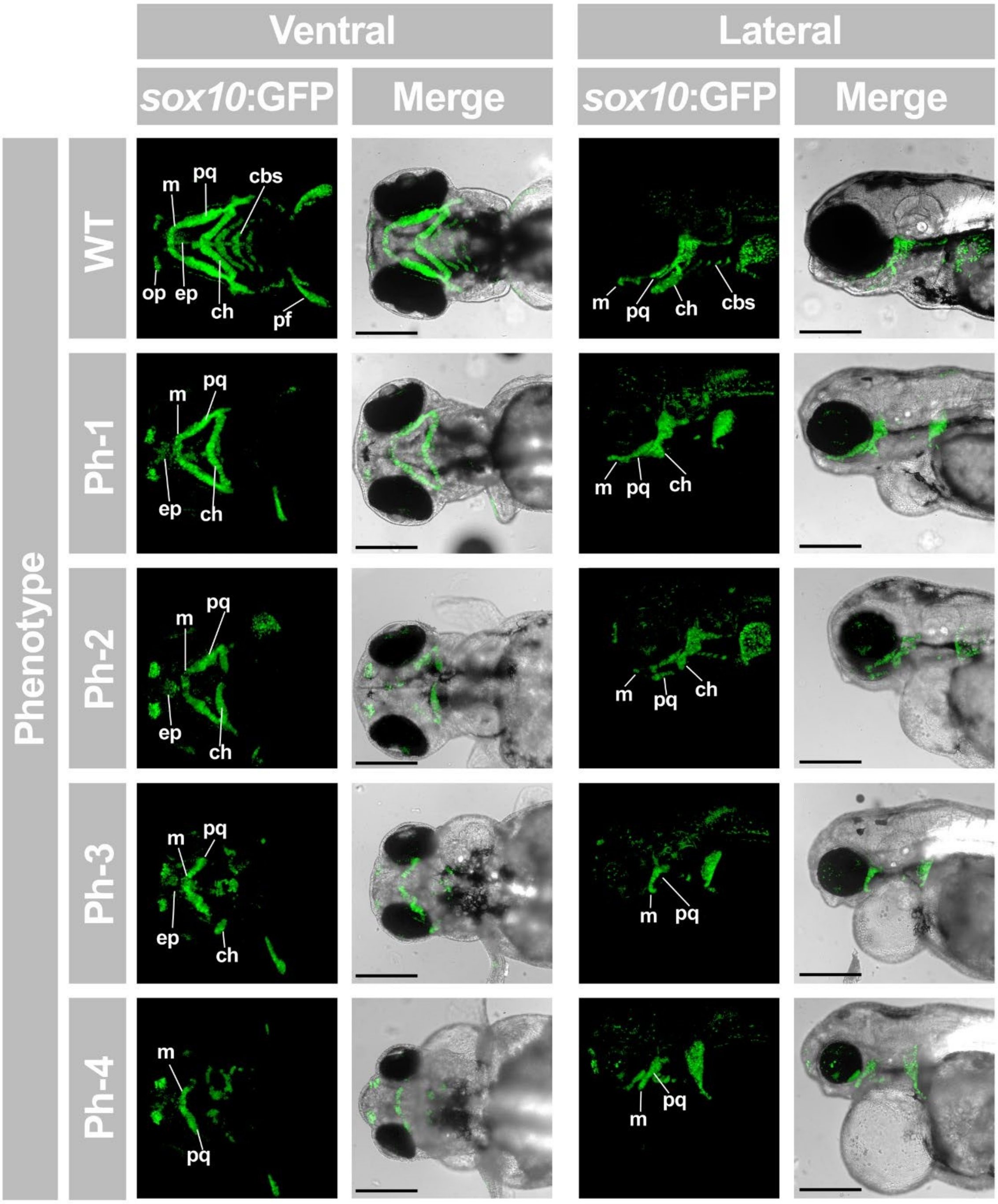


**Supplementary Figure 10. Characterization of jaw cartilague maldevelopment of *Gpatch3*$^{ATG}$ morphants at 96 hpf.** Reporter Tg(*sox10*:GFP) zebrafish embryos were microinjected with 2.0 ng of the *gpatch3*$^{ATG}$ MO. Merge of the maximum intensity projection of fluorescent signals (*sox10*:GFP) and transmitted light micrographs is also shown. cbs, ceratobranchials; ch, ceratohyal, m, Meckel's cartilage; ep, ethmoid plate; pq, palatoquadrate; op, olfactory pit; pf, pectoral fin. Scale bars represent 200 µm.
